# Propafenone-mediated gap junctional uncoupling results from aberrant connexin-43 trafficking

**DOI:** 10.1007/s43440-026-00845-7

**Published:** 2026-03-02

**Authors:** Encan Li, Najla Boujeddaine, Britt Mol, Emmy Penning de Vries, Marcel A.G. van der Heyden

**Affiliations:** 1 Department of Pharmacy, Tianjin First Central Hospital, Tianjin, China; 2 Department of Medical Physiology, Division of Heart & Lungs, University Medical Center Utrecht, Utrecht, The Netherlands; 3 Department of Medical Physiology, DH&L Yalelaan 50, Utrecht, 3584 CM The Netherlands

**Keywords:** Cx43, Propafenone, Accumulation, Metabolic coupling, Trafficking

## Abstract

**Background:**

Connexin-43 (Cx43) is the principal gap junction protein in the heart, mediating electrical coupling and ion exchange between cardiomyocytes to maintain synchronous contraction. Any disruption or malfunction of Cx43 can lead to arrhythmias and other cardiac issues. Understanding the functions and regulation of Cx43 is vital in both basic research and clinical contexts. Propafenone, a class Ic antiarrhythmic drug, has shown promise in rhythm control; however, its precise impact on cardiac cellular physiology, particularly regarding Cx43, remains incompletely understood. The present study investigated propafenone’s effects on Cx43 protein content, physiology, and underlying mechanisms in cell systems.

**Methods:**

Cell lines include human embryonic kidney HEK293 cells transfected with Cx43 (Ex-HEK); differentiated murine embryonic carcinoma EPI7 cells with an epithelioid morphology and visceral endoderm-like END2 cells, both endogenously expressing functional Cx43. Cx43 protein contents were determined by Western blot analysis, whereas immunofluorescence (IF) imaging was used to assess the subcellular localization of Cx43 proteins. Dye injections were used to gain insight into the effects of propafenone on Cx43 function.

**Results:**

Full-length Cx43 protein levels were dose-dependently increased after propafenone treatment and IF microscopy showed an intracellular accumulation of Cx43 protein, both on heterologously and endogenously expressed Cx43. Propafenone did not alter the Cx43 half-life, in contrast to the lysosomal inhibitor chloroquine. Finally, gap-junctional coupling was decreased by chronic propafenone treatment.

**Conclusion:**

We conclude that propafenone increases non-functional Cx43 protein content, resulting in its intracellular accumulation, as a side effect.

**Supplementary Information:**

The online version contains supplementary material available at 10.1007/s43440-026-00845-7.

## Introduction

Connexins (Cx) are basic protein units that contribute to the formation of gap junctions, specialized channels that directly connect neighboring cells [1]. Cardiomyocytes express three key Cx isoforms, which are essential to sustain a coordinated cardiac contraction, namely, Cx43, Cx40, and Cx45 [2]. Cx43 is the most abundant Cx isoform expressed in the heart, localized in ventricular and atrial cardiomyocytes, endothelial cells, smooth muscle cells, and fibroblasts [2, 3]. Studies have indicated that changes in the protein content and arrangement of Cx43 are linked to serious heart conditions, including arrhythmias, heart failure and even sudden cardiac death [3–9]. A key problem in these diseases is the loss of electrical coupling between cells [9, 10]. When Cx43 is dysfunctional, electrical signals spread more slowly between heart cells, drastically increasing the risk of arrhythmias. For instance, a 90% reduction in Cx43 can slow electrical conduction by approximately 50% [9, 11, 12]. Furthermore, Cx43 protein content is linked to the function of Nav1.5, a channel crucial for generating the heart electrical activity [13–16]. Therefore, the loss of Cx43 disrupts cell-to-cell coupling and makes the heart more vulnerable to arrhythmias.

Propafenone, a Class Ic antiarrhythmic drug, exerts its therapeutic effects primarily through the inhibition of sodium ion channels, playing a crucial role in restoring regular heart rhythms, especially in conditions like atrial fibrillation and flutter [17–19]. Propafenone therapeutic plasma concentrations in humans ranges from 0.53 to 5.28 µM mol L^− 1^ [21]. In human atrial tissues, propafenone concentrations are typically ten times higher than plasma concentrations [22]. As both propafenone and dysfunctional Cx43 contribute to a reduction in sodium current amplitude, exploring how propafenone influences Cx43 presents an interesting research direction.

Besides its current clinical use, we used propafenone as lead compound in developing more specific inward rectifier current activators [20]. As functional links between inward rectifier channels (i.e. K_IR_2.1) and Cx43 have been established [21], we also decided from this perspective to investigate a potential effect of propafenone on Cx43 protein content and function.

The present study suggests that propafenone leads to an increase in Cx43 protein content and causes its intracellular accumulation. Cx43 protein content and localization were determined by Western blot analysis and immunofluorescence techniques. Cx43 mediated metabolic coupling was tested by dye injections. The mechanism underlying the increase of Cx43 protein content induced by propafenone was investigated in this study. Understanding the intricate relationship between propafenone and Cx43 could potentially guide strategies for minimizing adverse effects while optimizing its antiarrhythmic properties, and assists in the development of inward rectifier current activators.

## Materials and methods

### Cell culture

Human embryonic kidney (HEK)-293 cells (ATCC, Accession Number: CRL-1573; [22]) and Ex-HEK cells (Human embryonic kidney (HEK)-293 cells stably expressing Cx43) (a kind gift from Nenad Bursac (Duke University, Durham, USA) [23, 24]) were cultured in Dulbecco’s Modified Eagles Medium (DMEM; Lonza, Breda, Netherlands) supplemented with 10% fetal bovine serum (FBS; Sigma-Aldrich, St. Louis, USA), 200 mM L-Glutamine (Lonza), and 10.000 U/mL penicillin-streptomycin (Lonza) at 37 °C with 5% CO_2_. Differentiated murine embryonic carcinoma EPI-7 cells [25] with an epithelioid morphology, and visceral endoderm-like END-2 cells [25] were cultured in DMEM/F12 (1:1; Gibco, Bleiswijk, the Netherlands) containing 10% fetal bovine serum (FBS; Sigma-Aldrich, St. Louis, USA), 200 mM L-Glutamine (Lonza), and 10.000 U/mL penicillin-streptomycin (Lonza) at 37 °C with 5% CO_2_. Cells for each time point were seeded on the same day, and drugs were added for the indicated time before harvesting all the samples. At the time of processing, cell confluency was 80–90%, 50–60%, and 95–100% for biochemical, immunofluorescent, and dye injection experiments respectively.

### Drugs

Chloroquine (CQ, Sigma, cat. No. C6628) was dissolved in sterile water at a concentration of 10 mM and stored at − 20 °C. Propafenone (racemic mixture; Sigma, cat. No. P4670) was dissolved in DMSO at a concentration of 100 mM and stored at − 20 °C until use. Cycloheximide (CHX, Sigma, cat. No. C7698) was dissolved in sterile water at 5 mg/mL, stored, and aliquoted at − 20 °C until use. Brefeldin A (BFA, MP Biomedicals, cat. No. 194802) was dissolved in DMSO at a concentration of 20 mg/mL and stored at − 20 °C until use.

### Western blot

Cells were lysed in Buffer D (20 mM HEPES, 125 mM NaCl, 10% glycerol, 1 mM EGTA, 1 mM dithiothreitol, 1 mM EDTA, and 1% Triton X-100 (pH 7.6)) supplemented with 4 µg/mL aprotinin and 0.2 mM phenylmethylsulphonyl fluoride (PMSF). Protein Lysate (24 µg) was separated by 10% SDS-PAGE and blotted onto a nitrocellulose membrane (Bio-Rad Laboratories, Veenendaal, The Netherlands). Ponceau staining was used as a loading control for subsequent quantification. Blots were blocked for 60 min with 5% bovine serum albumin (BSA) dissolved in Tris-buffered saline/Tween 20 (20 mM Tris-HCl (pH 8.0), 150 mM NaCl, 0.05% (v/v) Tween-20). For protein detection, the blot was incubated with an anti-Cx43 antibody (1:400, BD Biosciences, Lexington, KY, USA, cat. no. 610062). Subsequently, peroxidase-conjugated Goat anti-mouse (1:7000, Jackson ImmunoResearch, West Grove, PA, USA, cat. no. 115-035-003) was applied as the second antibody. Final detection was performed using the Standard ECL procedure (Amersham Bioscience, Buckinghamshire, UK). Quantification was performed with Image Lab software version 6.1 (Bio-Rad Laboratories, Veenendaal, The Netherlands).

### Immunofluorescence microscopy

EPI-7 and END-2 cells were cultured on 0.1% gelatin coated ∅ 15 mm glass coverslips. The cells were treated with Propafenone (10, 40, 50 µM) or CQ (10 µM) for 24 h. Then the coverslips were washed with PBS, fixated with 3% paraformaldehyde, permeabilized with 0.5% Triton X-100 (in PBS), quenched with PBS/glycine (50 mM), and incubated twice with NET-gel (0.25% gelatin, 50 mM Tris-Cl, 150 mM NaCl, 4 mM EDTA, 0.05% Igepal, 0.01% NaN_3_, pH 7.4). Primary antibodies used were Cx43 (1:250, BD Biosciences) and zonula occludens (ZO-1, 1:150, Invitrogen, Rockford, IL, USA, cat. no. 61-7300). After washing the cells, coverslips were incubated with secondary antibodies: donkey anti-mouse (Green, 1:100; Jackson ImmunoResearch, West Grove, PA, USA, cat. no. 715-095-151) and donkey anti-rabbit (Alexa Red, 1:350; Jackson ImmunoResearch, cat. no. 711-585-152). Cell nuclei were stained with 4’, 6-diamindino-2-phenylindole (DAPI; Molecular Probes, Leiden, Netherlands). Coverslips were mounted with Vectashield (Vector Laboratories, Burlingame, USA) and imaged using a microscope (Nikon Instruments Europe B.V., Amsterdam, The Netherlands) equipped with a 60x ⋅ oil immersion objective (CAIRN research, Faversham, UK). Imaging and linescan analysis was performed using Metamorph software (Version 7.10.5.476).

### Time effect experiment

EPI-7 and END-2 cells were cultured overnight in ∅ 60 mm dishes. Then cells were treated with propafenone (50 µM). Protein lysates were harvested 0, 2, 4, 6, 8, or 24 h following addition of the drugs. Non-transfected cells (NT) were used as a negative control. Cell lysates were prepared and processed as indicated in Section “Western blot”.

### Dye injections

EPI-7 and END-2 cells were cultured in ∅ 35 mm glass-bottom dishes overnight. The cells were then treated with propafenone (50 µM) or CQ (10 µM) for 24 h. The culture medium was replaced by the FluoroBrite DMEM Live Cell Fluorescence Imaging Medium (Gibco, Grand Island, NY, USA). Micropipettes (tip diameter of 0.2 μm) were made with a Sutter P-2000 puller (HEKA Elektronik, Lambrecht, Germany). The pipettes were filled with 4% Lucifer Yellow solution in 150 mmol/L LiCl. The dye-containing micropipettes were inserted in selected cells in clusters of at least 20 visually connected cells. The dye was allowed to diffuse out of the pipette into the impaled cell and its adjacent cells for 2 min. The pipette was then retracted, and the cells were viewed with epifluorescent illumination (excitation 420–490 nm, emission > 520 nm). Connected cells became fluorescent and were counted.

### Cycloheximide assay

Ex-HEK cells were seeded in ∅ 60 mm dishes. After 24 h, cells were left untreated (control) or were treated with 50 µM propafenone or 10 µM CQ for 24 h. Chloroquine (CQ), a known inhibitor of lysosomal function, was used as a positive control [22, 26]. 200 µg/mL CHX was added during the last phases (2, 4, 6, and 8 h) of the 24 h treatment period. Non-transfected cells (NT) were used as a negative control. Cell lysates were prepared and processed as indicated in Section “Western blot”.

### BFA assay

Ex-HEK cells were seeded in ∅ 60 mm dishes. After 24 h, cells were left untreated (control) or treated with BFA (2 µg/mL) or BFA (2 µg/mL) combined with propafenone (50 µM) for 4 h. Non-transfected cells (NT) were used as a negative control. Cell lysates were prepared and processed as indicated in Section “Western blot”.

### Statistics

**Fig. 1:**
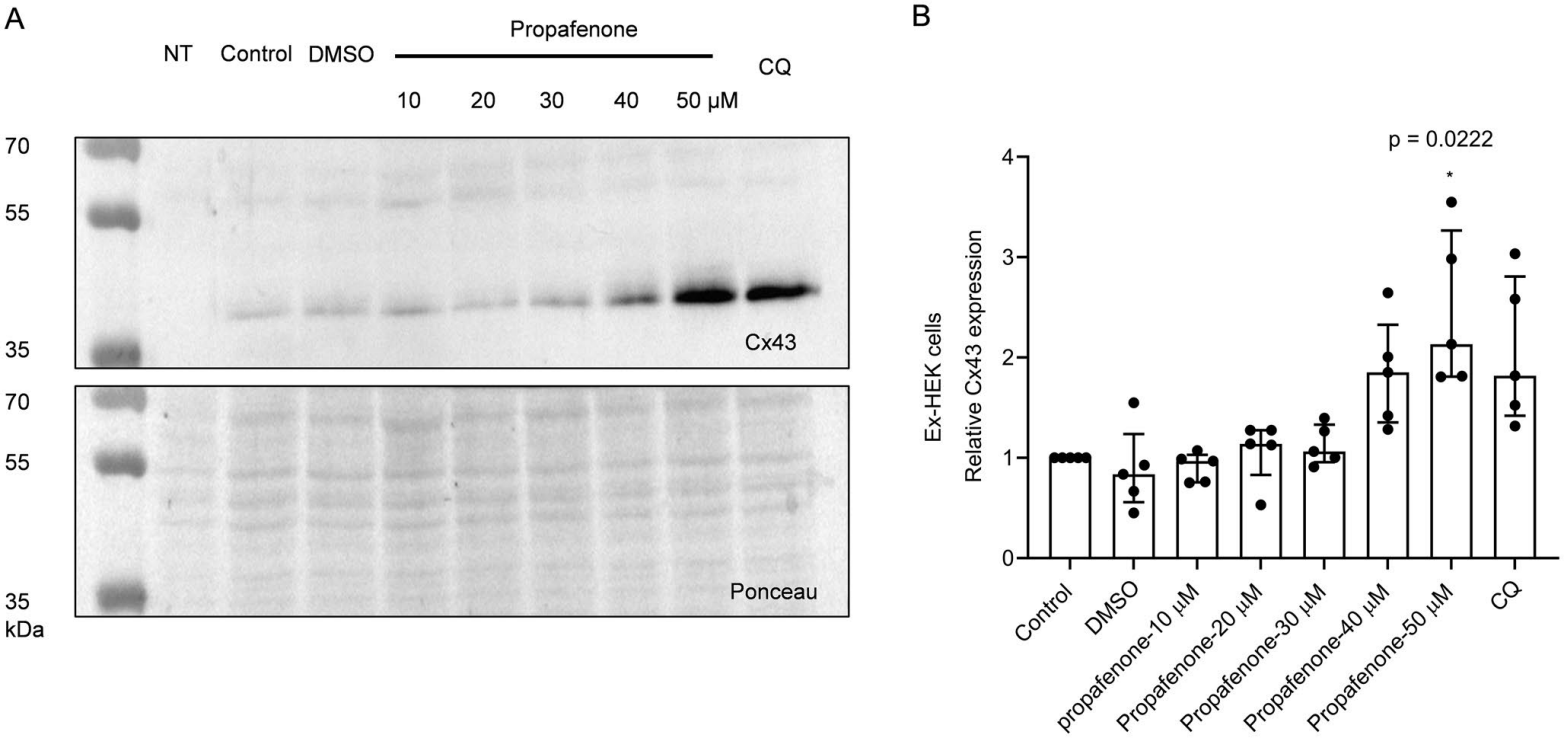
Propafenone increases Cx43 protein content in Ex-HEK cells. Western blot analysis of Cx43 protein in Ex-HEK cells. Cells were treated with different concentrations of propafenone (10, 20, 30, 40, 50 µM) for 24 h (*n* = 5, number of independent experiments). Non-transfected (NT) cells served as a negative control. (**A**) Representative Western blot membranes. (**B**) Quantification of Cx43 protein levels in control and treated cells. The control protein level was set at 1 after correcting for loading. Ponceau staining was used as a loading control. The Kruskal–Wallis test was used to determine statistical significance, followed by Dunn’s post hoc test to correct for multiple comparisons. Data are expressed as median with interquartile range. *p < 0.05 vs. Control. Abbreviations: CQ = Chloroquine, Cx43 = Connexin-43, DMSO = Dimethyl sulfoxide, NT = Non-transfected cells

**Fig. 2:**
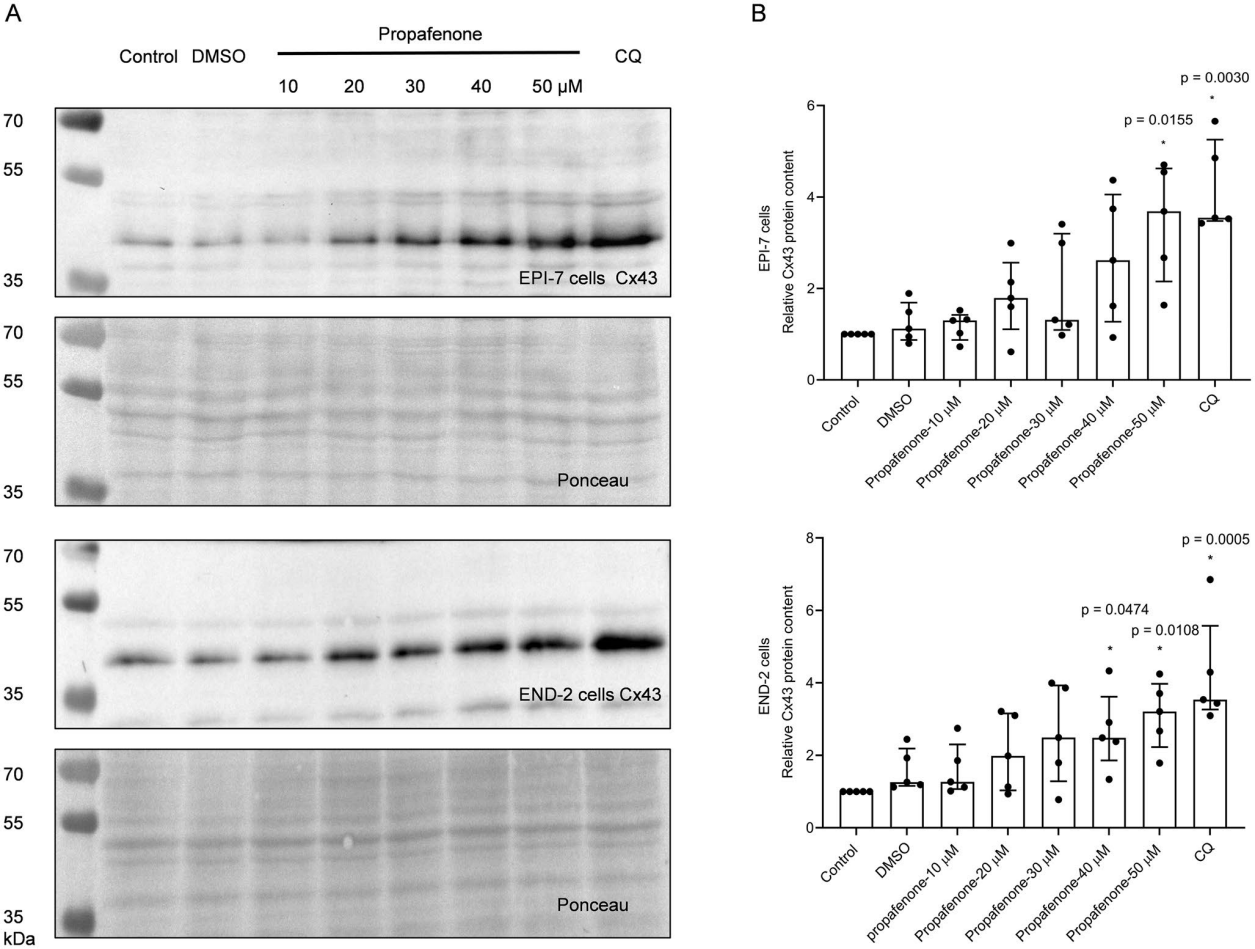
24 h propafenone treatment increases Cx43 protein content in EPI-7 and END-2 cells. Western blot analysis of Cx43 protein in EPI-7 and END-2 cells. Cells were treated with different concentrations of propafenone (10, 20, 30, 40, 50 µM) for 24 h (*n* = 5, number of independent experiments). (**A**) Representative Western blot membranes. (**B**) Quantification of Cx43 protein levels in control and treatment cells. The control protein level was set at 1 after correcting for loading. Ponceau staining was used as a loading control. The Kruskal–Wallis test was used to determine statistical significance, followed by Dunn’s post hoc test to correct for multiple comparisons. Data are expressed as median with interquartile range. *p < 0.05 vs. Control. Abbreviations: CQ = Chloroquine, Cx43 = Connexin-43, DMSO = Dimethyl sulfoxide

Data are expressed as either median with interquartile range (Figs. 1, 2, 4, 5 and 7) or mean ± SD (Fig. 6). The sample size subjected to statistical analysis was at least five experiments per condition (*n* = 5), where n is the number of independent values. The obtained results were analyzed with the use of the GraphPad Prism version 10 (GraphPad Software, San Diego, CA, USA). In western blots, signal intensity variability between experiments is mainly determined by antibody binding efficiency and ECL exposure time and other technical variabilities. Therefore, treatment effects can only be compared to the control condition on the same western blot. To enable direct comparison between control and treatment groups from subsequent biological replicates, data from control groups were normalized to 1 with no variance (SD = 0) (Figs. 1, 2, 4, 6A and 7). For Fig. 6B and C, data from 24 h CQ or propafenone treatment groups (without CHX) were normalized to 1 (SD = 0). Log-transformation of the y-values was applied to linearize the Cx43 degradation curves in Fig. 6D. The Shapiro–Wilk test indicated that all the data did not follow a normal distribution; therefore, nonparametric tests were applied. Specifically, the Kruskal–Wallis test was used to determine statistical significance, followed by Dunn’s post hoc test to correct for multiple comparisons.

Data were considered significant when the *p*-value was less than 0.05 (*p < 0.05). Although the raw data in Fig. 6 did not follow a normal distribution, the residuals of the fitted regression were confirmed to be normally distributed by the Shapiro-Wilk test, thereby justifying presentation as mean ± SD.

## Results

### Propafenone induces intracellular accumulation of Cx43

**Fig. 3:**
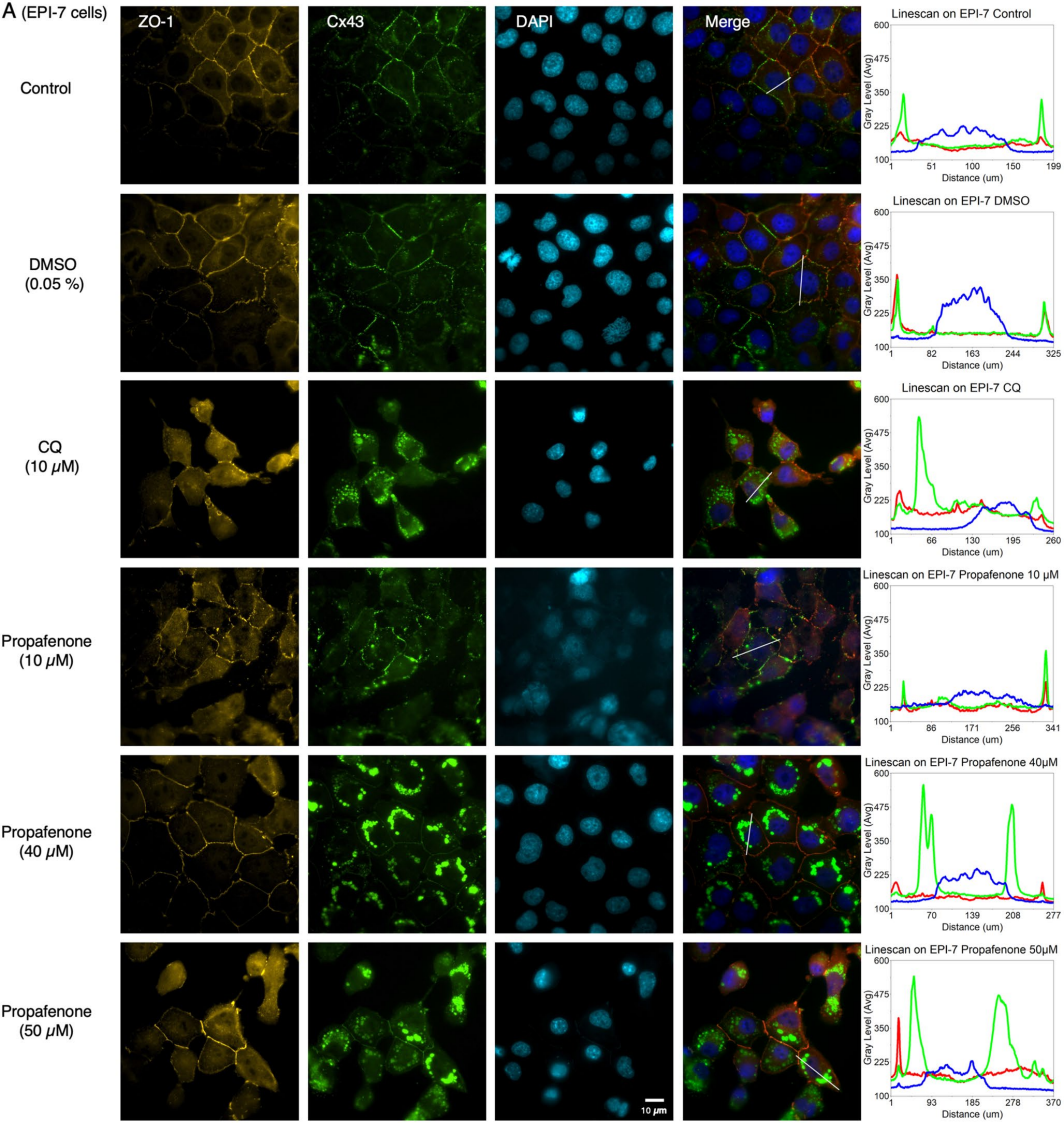

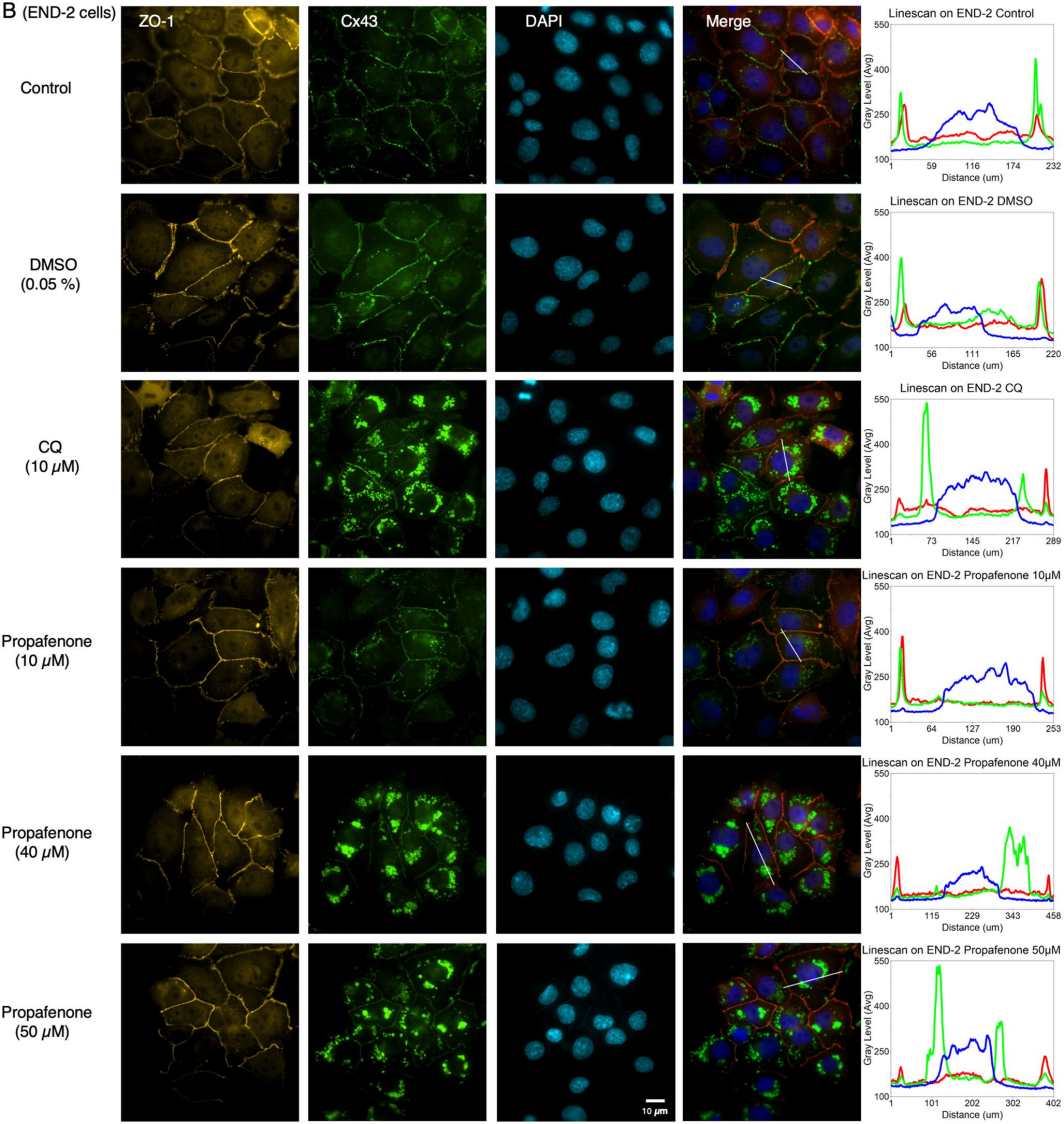
Propafenone induces intracellular accumulation of Cx43 protein. Propafenone induces Cx43 protein clustering in EPI-7 (**A**) and END-2 cells (**B**). Control groups included cells without treatment, cells treated with DMSO (0.05%) for 24 h; CQ groups indicated cells treated with CQ (10 µM) for 24 h and were used as a positive control. Treatment groups indicate cells treated with Propafenone (10, 40, 50 µM) for 24 h. Images were taken at 60 × magnification. 488 nm laser light was used to visualize Cx43. Cx43 was detected by Cx43 antibody (green), membrane staining by ZO-1 antibody (red), and the nuclei with DAPI (blue). The graph Linescan of selected regions containing cell-cell contact is indicated in the merged pictures. Results of the linescan recordings are given in the right panels. The linescan recordings show the spatial localization of Cx43 (green) relative to the nucleus (blue) and the cell membrane (red). The position of the Cx43 signal along the line indicates whether it is closer to the nucleus or the membrane. The scale bar indicates 10 μm. Abbreviations: CQ = Chloroquine, Cx43 = Connexin-43, DAPI = 4’,6-Diamidino-2’-phenylindole, DMSO = Dimethyl sulfoxide, ZO-1 = Zonula occludens-1

The Kruskal-Wallis test identified a statistically significant overall effect of propafenone on increasing Cx43 protein content (*p* < 0.05). Dunn’s post hoc test for multiple comparisons confirmed that this increase was significant at a concentration of 50 µM in Ex-HEK cells (H(7) = 28.39, *p* = 0.0222) (Fig. 1A, B). To confirm this finding in a model with endogenous Cx43 expression, the experiments were repeated in EPI-7 and END-2 cells. Similarly, the Kruskal-Wallis test showed a significant overall effect, with Dunn’s post hoc analysis revealing a significant increase in Cx43 protein content in both EPI-7 (H(7) = 22.10, *p* = 0.0155) and END-2 (H(7) = 21.15, *p* = 0.0108) cells (Fig. 2A, B). Qualitative immunofluorescence microscopy in EPI-7 and END-2 cells demonstrated that treatment with propafenone (40 and 50 µM) resulted in an intracellular accumulation of Cx43 protein, characterized by the formation of clusters (Fig. 3).

### Rapid upregulation of Cx43 overall protein content by propafenone within 4 h

**Fig. 4:**
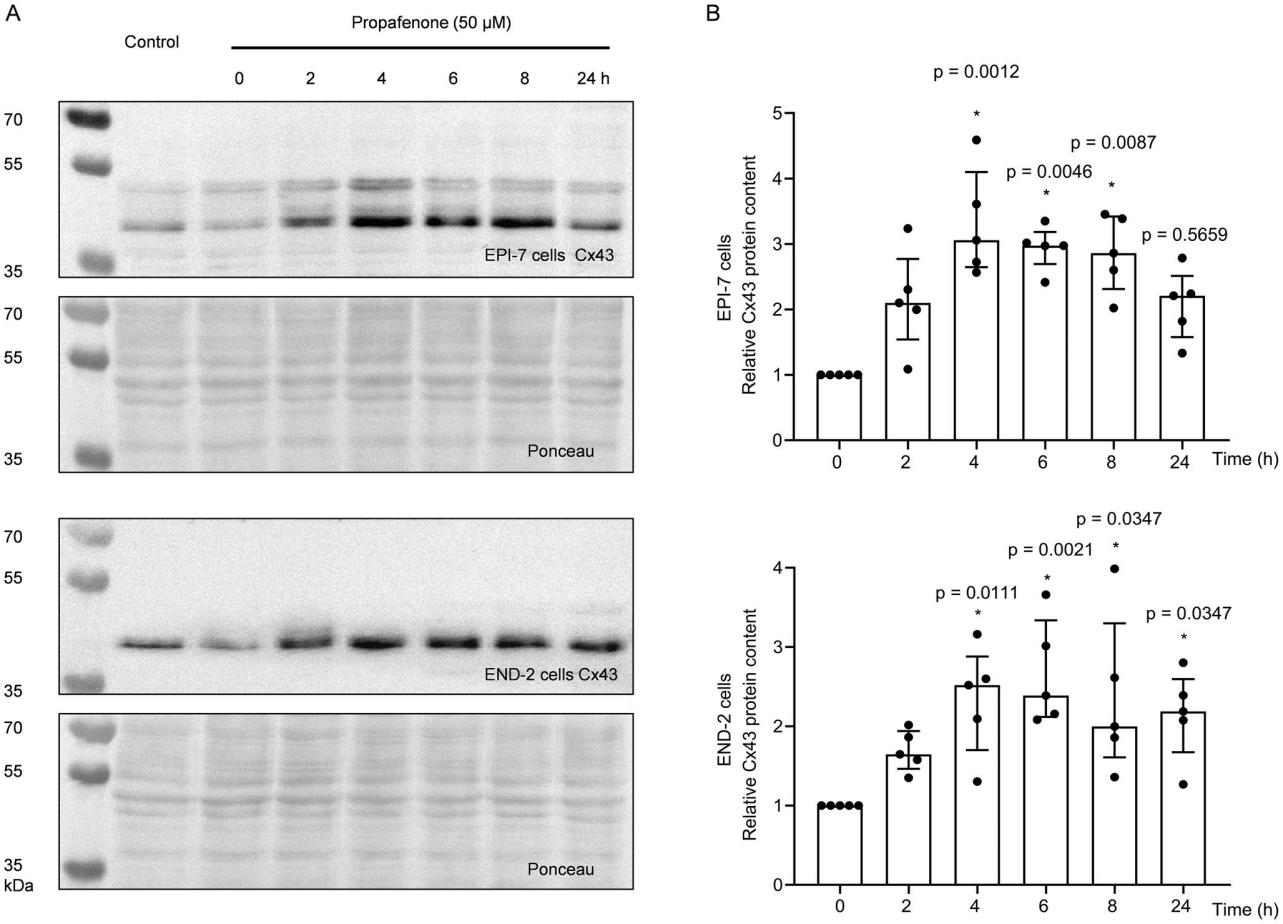
Propafenone rapidly upregulates the overall content of Cx43 protein. Western blot analysis of Cx43 protein content in EPI-7 and END-2 cells. Cells were treated with propafenone (50 µM) for different periods (0, 2, 4, 6, 8, 24 h) (*n* = 5, number of independent experiments). (**A**) Representative Western blot membranes. (**B**) Quantification of Cx43 protein levels in control and treated cells. The control protein level was set at 1 after correcting for loading. Ponceau staining was used as a loading control. The Kruskal–Wallis test was used to determine statistical significance, followed by Dunn’s post hoc test to correct for multiple comparisons. Data are expressed as median with interquartile range. *p < 0.05, 1 vs. t = 0 h. Abbreviations: Cx43 = Connexin-43

To test the time effect of propafenone on Cx43 protein content, Western blots were performed on EPI-7 and END-2 cell lysates after treating the cells with propafenone (50 µM) for 0, 2, 4, 6, 8, or 24 h. In the 0 h treatment group, propafenone was added and removed immediately. The Kruskal-Wallis test and subsequent Dunn’s post hoc comparisons indicated that in EPI-7 cells, propafenone (50 µM) induced a significant increase in Cx43 levels at the 4-hour time point compared to the 0-hour control (H(5) = 19.27, *p* = 0.0012), with this time point representing the peak observed effect (Fig. 4). A similar significant increase was also observed in END-2 cells at 4 h (H(5) = 16.60, *p* = 0.0111) (Fig. 4). We have not established drug concentration in cell medium and cells after 24 h and thus have no information on propafenone T_1/2_ in vitro.

### Propafenone induces a decline in gap junction coupling

**Fig. 5:**
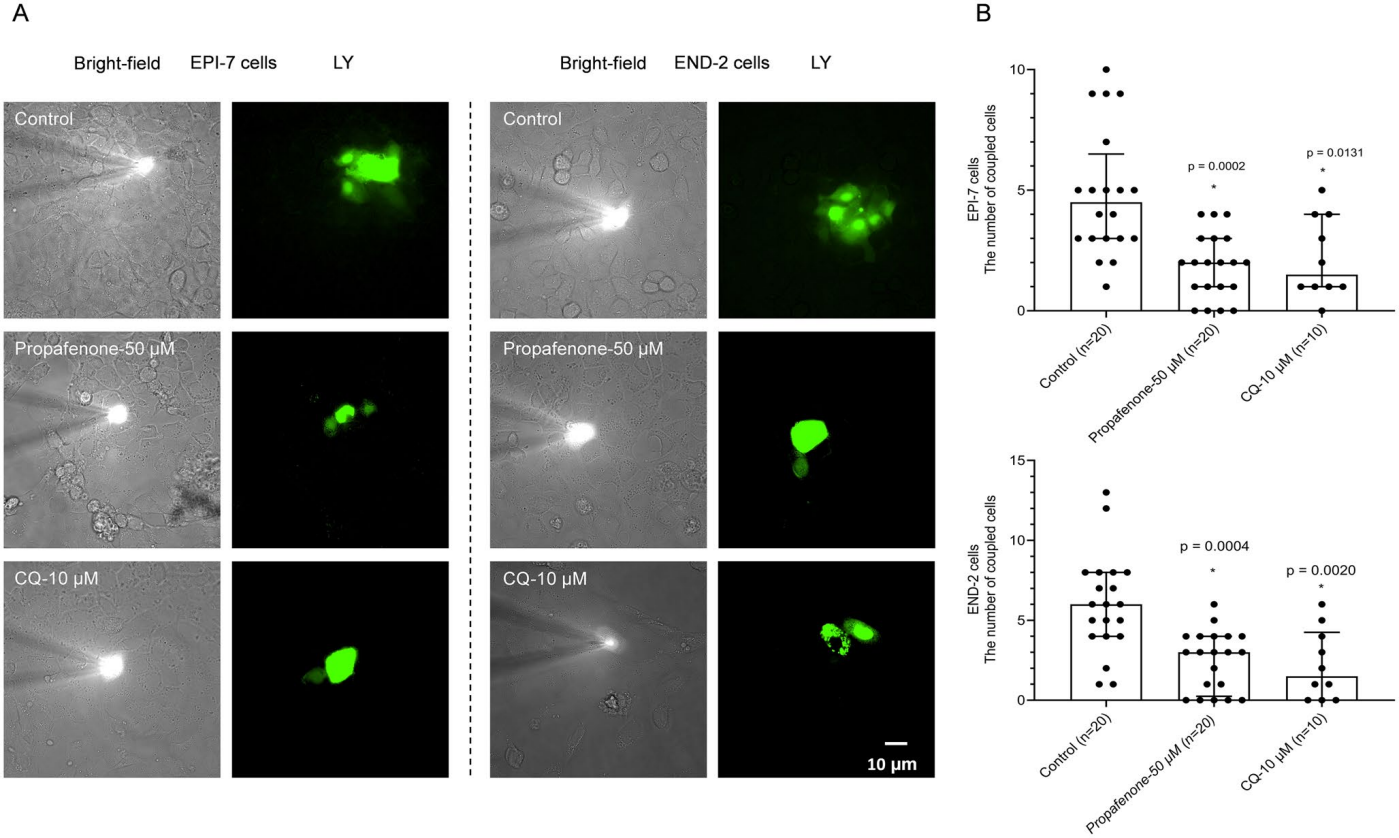
Propafenone induces a decline in gap junction coupling. EPI-7 and END-2 cells were treated with 50 µM propafenone or 10 µM CQ for 24 h. One test cell, within a large cluster of cells (> 20), was injected with 4% Lucifer Yellow. The dye was allowed to spread for two minutes and was subsequently visualized by a GFP camera. Subsequently, phase-contrast pictures from the same area were taken. Injected cells are indicated by the tip of the micropipette. (**A**) Microscopy fields depict the injected cells and those that were coupled when Lucifer Yellow was injected. Images were taken at 60 × magnification. The scale bar indicates 10 μm. (**B**) Quantification of the number of coupled cells in control, propafenone-, and CQ-treated conditions. The analysis comprises 20 microinjections per control and propafenone group, and 10 microinjections for CQ group. The Kruskal–Wallis test was used to determine statistical significance, followed by Dunn’s post hoc test to correct for multiple comparisons. Data are expressed as median with interquartile range. *p < 0.05 vs. Control. Abbreviations: CQ = Chloroquine, Cx43 = Connexin-43, LY = Lucifer yellow

Dye microinjections were performed in EPI-7 and END-2 cells using the gap junction-permeable fluorescent dye Lucifer Yellow [27]. The Kruskal-Wallis test with Dunn’s post hoc analysis revealed that dye spreading was significantly reduced after 24-hour treatment with propafenone (50 µM) compared to the control group in both EPI-7 (H(2) = 16.66, *p* = 0.0002) and END-2 (H(2) = 17.63, *p* = 0.0004) cells (Fig. 5). A similar significant inhibition was observed with chloroquine (CQ, 10 µM) in EPI-7 (H(2) = 16.66, *p* = 0.0131) and END-2 (H(2) = 17.63, *p* = 0.0020) cells (Fig. 5). This functional impairment in gap junction coupling correlates with the observed intracellular localization of Cx43, underscoring that proper membrane localization is critical for Cx43 functionality. This propafenone-induced dysfunction reveals a significant side effect, suggesting that therapeutic strategies aimed at alleviating Cx43 intracellular accumulation could represent a novel future approach.

### Propafenone lacks an impact on the half-life of Cx43 but potentially enhances ER protein synthesis

**Fig. 6:**
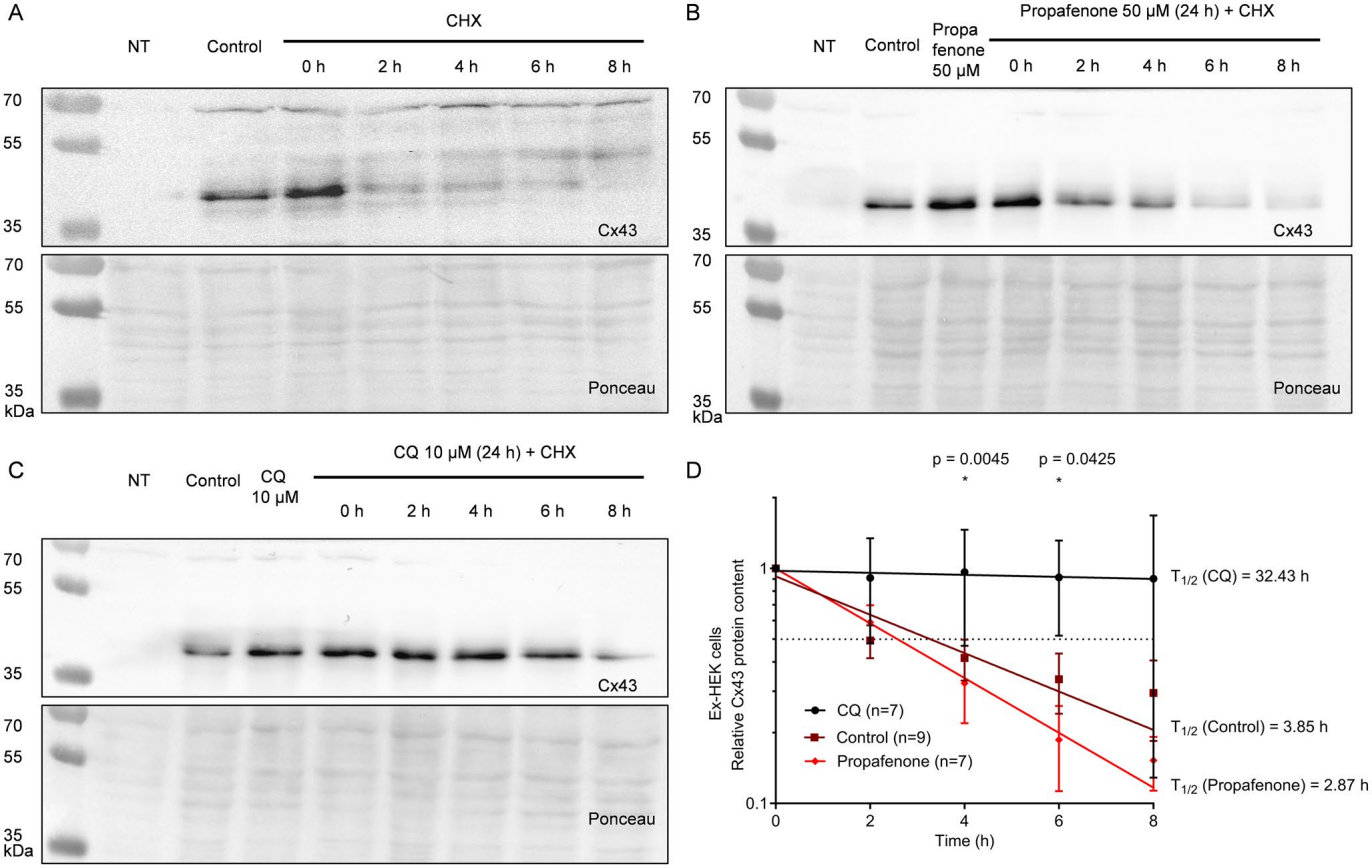
CHX assay of Cx43 protein degradation in Ex-293 cells. (**A**) Example of Cx43 protein degradation after exposure to 200 µg/mL CHX for different time intervals. (**B**) Example of Cx43 protein degradation in cells that were treated with propafenone (50 µM) for 24 h. 200 µg/mL CHX was added for different periods before lysates were prepared at t = 24 h. (**C**) Example of Cx43 protein degradation in cells that were treated with CQ (10 µM) for 24 h. 200 µg/mL CHX was added for different periods before lysates were prepared at t = 24 h. (**D**) Quantification of CHX assays to depict normalized Cx43 protein content vs. timed CHX treatment with or without propafenone or CQ treatment. The dotted line indicates 50% of the initial normalized Cx43 signal. Non-transfected cells (NT) were used as a negative control. The Kruskal–Wallis test was used to determine statistical significance, followed by Dunn’s post hoc test to correct for multiple comparisons. Data are expressed as mean ± SD.* *p* < 0.05 when CQ vs. Control. Abbreviations: CHX = cycloheximide, CQ = Chloroquine, Cx43 = Connexin-43, NT = Non-transfected cells

CQ treatment significantly stabilized Cx43, as evidenced by a prolonged half-life (T_1/2_= 32.43 h vs. 3.85 h) and significantly elevated protein levels at both 2 h (H(2) = 8.16, *p* = 0.0129) and 4 h (H(2) = 14.50, *p* = 0.0067) compared to the control, based on the Kruskal-Wallis test followed by Dunn’s post hoc analysis (Fig. 6A, C, D). In contrast, post hoc comparisons showed no significant difference in Cx43 half-life between the propafenone-treated group and the control group (T_1/2_ = 2.87 h vs. 3.85 h) (Fig. 6A, B, D). This indicates that, unlike CQ, propafenone does not impair the degradation pathway of Cx43 protein.

**Fig. 7:**
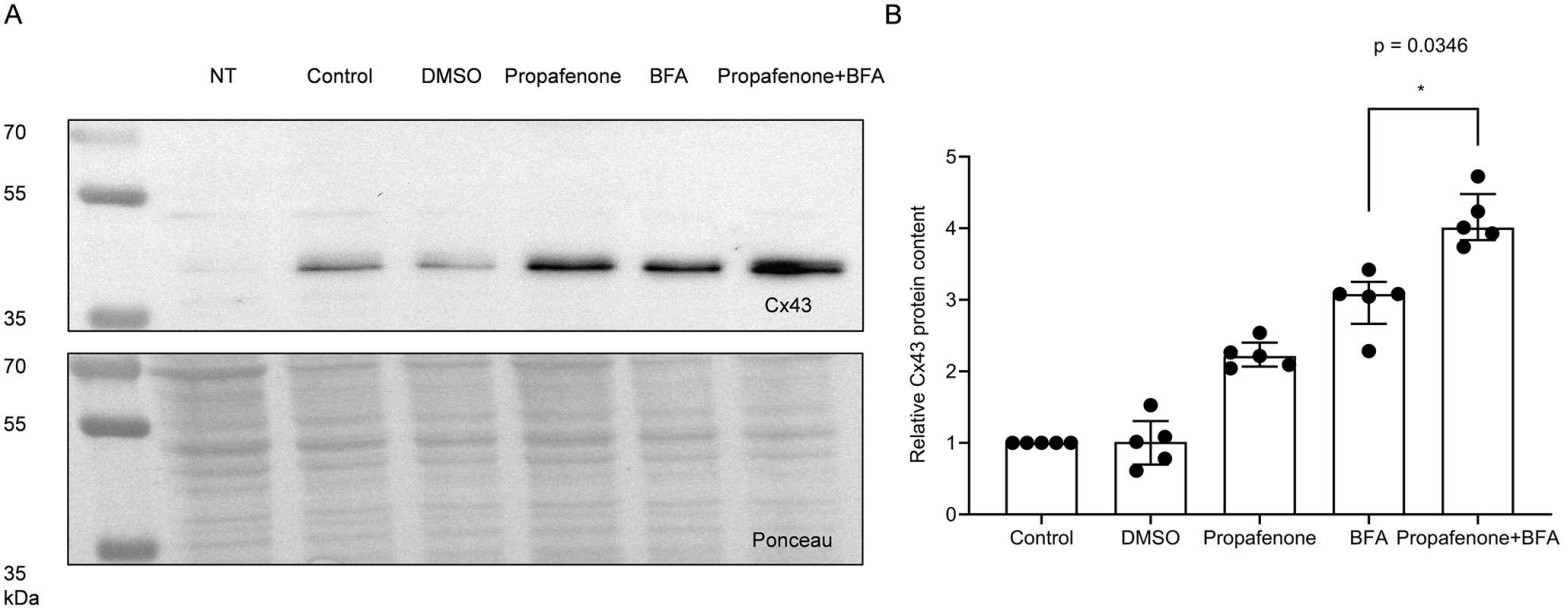
Propafenone enhances Cx43 protein synthesis. Western blot analysis of Cx43 protein in Ex-HEK cells. Cells were treated with BFA (2 µg/mL) or BFA (2 µg/mL) combined with propafenone (50 µM) for 4 h (*n* = 5, number of independent experiments). Non-transfected (NT) cells were used as a negative control. (**A**) Representative Western blot membranes. (**B**) Quantification of Cx43 protein levels in control and treated cells. The control protein level was set at 1 after correcting for loading. Ponceau staining was used as a loading control. The Kruskal–Wallis test was used to determine statistical significance, followed by Dunn’s post hoc test to correct for multiple comparisons. Data are expressed as median with interquartile range.*p < 0.05, BFA combined with propafenone vs. BFA alone. Abbreviations: BFA = Brefeldin A, Cx43 = Connexin‑43, DMSO = Dimethyl sulfoxide

To investigate the impact of propafenone on the endoplasmic reticulum (ER), Ex-HEK cells were treated with propafenone together with Brefeldin A (BFA) or BFA only. BFA blocks the forward trafficking of Cx43 and disrupts Golgi organization at 2 µg/mL [28–31]. Statistical analysis using the Kruskal-Wallis test with Dunn’s post hoc test demonstrated that Cx43 protein content was significantly higher in cells treated with both propafenone and BFA compared to those treated with BFA alone (H(4) = 21.96, *p* = 0.0346; Fig. 7). This result suggests that propafenone may enhance the synthesis of Cx43 protein within the ER.

## Discussion

Propafenone is a widely used drug for treating cardiac arrhythmias by its modulating effect on sodium ion channels [17–19, 32]. Cx43 plays a crucial role in coordinating electrical activity between neighboring cells, which influences cardiac conduction and rhythm [1, 10]. However, a decrease in Cx43 protein content is associated with a decline in Na_V_1.5 channel protein content, reducing sodium current amplitude [13–16]. Although the role of propafenone in rhythm control is well established, its impact on Cx43 is less understood. This study found an intracellular accumulation in Cx43 proteins after exposure to propafenone. This unexpected accumulation raises questions about the underlying molecular mechanisms governing this phenomenon.

This study provides systematic experimental evidence that propafenone upregulates Cx43 protein content primarily by enhancing protein synthesis rather than inhibiting degradation pathways. This conclusion is supported by several key findings: First, protein stability assays confirmed that propafenone treatment did not alter the half-life of Cx43, effectively ruling out involvement of proteasomal, lysosomal, or autophagic degradation pathways. More importantly, experiments using the transport inhibitor BFA revealed an instructive phenomenon: when propafenone was combined with BFA, Cx43 protein contents were significantly enhanced compared to BFA treatment alone. BFA blocks Golgi function and inhibits protein maturation and transport. Treatment with 2 µg/mL of BFA for 4 h resulted in less than 20% of Cx43 proteins being able to anchor to the cell membrane, leading to the accumulation of numerous proteins in the endoplasmic reticulum apparatus [30, 33]. The fact that propafenone still further increased Cx43 levels under these conditions strongly suggests that its site of action lies within the protein synthesis machinery. It has been reported that Cx43 overexpression can induce endoplasmic reticulum stress (ERS) by disturbing the balance between the protein-folding burden and the ER’s processing capacity [34–37]. However, in preliminary experiments, propafenone was unable to induce protein content of six different ERS markers in Ex-HEK cells, in contrast to BFA (Supplemental Fig. S1). This might indicate that the increase in Cx43 protein content mediated by propafenone does not provoke a detectable ER stress response. The apparent absence of ERS activation suggests that alternative mechanisms may account for the enhanced Cx43 levels. These could include that propafenone interacts directly with components of the vesicular transport machinery, facilitating efficient export of Cx43 from the ER without exceeding its folding capacity. Such a mechanism would regulate increased Cx43 synthesis to avoid ERS, positioning propafenone as a modulator that enhances connexin synthesis while maintaining ER homeostasis.

Microtubules are known to mediate Cx43 transport, and altered α-tubulin detyrosination (Glu) can impair trafficking [38–41]. First findings show that propafenone did not affect Glu-α-tubulin levels (Supplemental Fig. S2) which suggests that microtubule detyrosination is not responsible for the observed Cx43 accumulation. However, other post-translational modifications, such as α-tubulin acetylation, which influences cargo transport and has been linked to Cx43 remodeling in cardiomyopathy, deserve further investigation [38, 42].

Beyond synthesis and microtubule-dependent transport, endocytosis represents another potential regulatory way [43]. Although certain kinases regulate Cx43 internalization [43–46], no evidence currently links propafenone to these pathways. The parallel intracellular accumulation of Cx43 and K_IR_2.1 channels observed in our and previous studies suggests a shared trafficking disruption, possibly in backward trafficking [22, 47–51]. Studies also indicate a close interaction between Cx43 and mitochondrial function [52–55]. However, the exact molecular mechanisms responsible for the intracellular accumulation of Cx43 protein due to propafenone are unclear. Further research is needed to clarify this.

Intracellular protein accumulation can be cytotoxic either by gaining toxic activity or by losing biological function [56]. Intracellular accumulation of Cx43, shown here, leads to a decrease in cell coupling and may have consequences on cardiac electrophysiology. Disruption of gap junction properties could affect impulse propagation, potentially leading to arrhythmias or conduction disturbances [2, 57]. Overexpression of Cx43 also shows detrimental in the developing heart [58]. The consistent observation of propafenone-induced Cx43 accumulation and trafficking impairment across multiple cell models-including Ex-HEK cells with heterologous expression, and EPI-7 and END-2 cells with endogenous Cx43—strengthens the robustness of our conclusions. This consistency across systems with differing Cx43 sources and protein content indicates that the effect is a general cellular response. To further strengthen these findings and potential implications for cardiac electrophysiology, follow-up studies using adult cardiomyocytes are necessary. Cx43 is also present in the central nervous system and various types of cancer cells [59, 60]. Changes in the expression and accumulation of Cx43 have been observed in neurological disorders and implicated in cancer progression [60, 61]. Therefore, we conclude that intracellular accumulation of Cx43 is one of propafenone’s side effects. This knowledge could influence treatment strategies, especially in patients with pre-existing conditions associated with Cx43 dysfunction, as for example in arrhythmogenic cardiomyopathy [62, 63]. Additionally, it may prompt the development of more targeted antiarrhythmic therapies that minimize unintended effects.

This study comes with several limitations. Although total Cx43 levels were detected at whole cell level, Cx43 cellular localization was assessed by immunofluorescence microscopy, and gap-junctional function was addressed by dye-coupling, we did not quantify Cx43 protein levels at the plasma membrane. Subsequent studies, using for example, plasma membrane biotin labeling of Cx43 in response to propafenone would be informative in this aspect. Secondly, although Cx43 expression was found to be increased in response to propafenone, unrelated to a change in Cx43 half-life, we did not study Cx43 mRNA content in response to propafenone. Therefore, we cannot exclude that propafenone increases Cx43 mRNA synthesis (although propafenone effects were observed on both endogenous Cx43 and ectopic Cx43 driven by a viral promoter), or mRNA stability. Again subsequent studies exploring the potential effect of propafenone on Cx43 transcription efficacy and mRNA stability on endogenous Cx43 in relevant cell types (i.e. adult ventricular and atrial cardiomyocytes) will be of interest.

## Supplementary Information

Below is the link to the electronic supplementary material.

Supplementary Material 1

Supplementary Material 2

Supplementary Material 3

## Data Availability

The datasets generated during and/or analysed during the current study are available from the corresponding author upon reasonable request.

## Abbreviations

BFA: Brefeldin A
Bip: Immunoglobulin heavy chain binding protein
CHOP: CCAAT-enhancer-binding protein homologous protein
CHX: Cycloheximide
CQ: Chloroquine
Cx: Connexin
DAPI: 4',6-Diamidino-2'-phenylindole
DMSO: Dimethyl sulfoxide
Erol-Lα: Endoplasmic reticulum oxidoreductin-1-Like αERS: Endoplasmic reticulum stress
FBS: Fetal bovine serum
IRE1α: Inositol-requiring enzyme type 1α
K_IR_2.1: Inward rectifier potassium channel
NT: Non-transfected cells
PDI: Protein disulfide isomerase
PERK: Protein kinase-like endoplasmic reticulum kinase pathways
ZO-1: Zonula occludens-1
