# Supplementary material for: Propafenone-mediated gap junctional uncoupling results from aberrant connexin-43 trafficking
Source: Pharmacol Rep. 2026 Mar 2;78(3):849–61. doi: 10.1007/s43440-026-00845-7 (PMC13275528; doi:10.1007/s43440-026-00845-7)

Western Blotting Original Data

Fig-1, CX-43 Expression-Bands (Ex-HEK Cells)

Propafenone

Marker NT Con DMSO CQ

10 20 30 40 50 μM

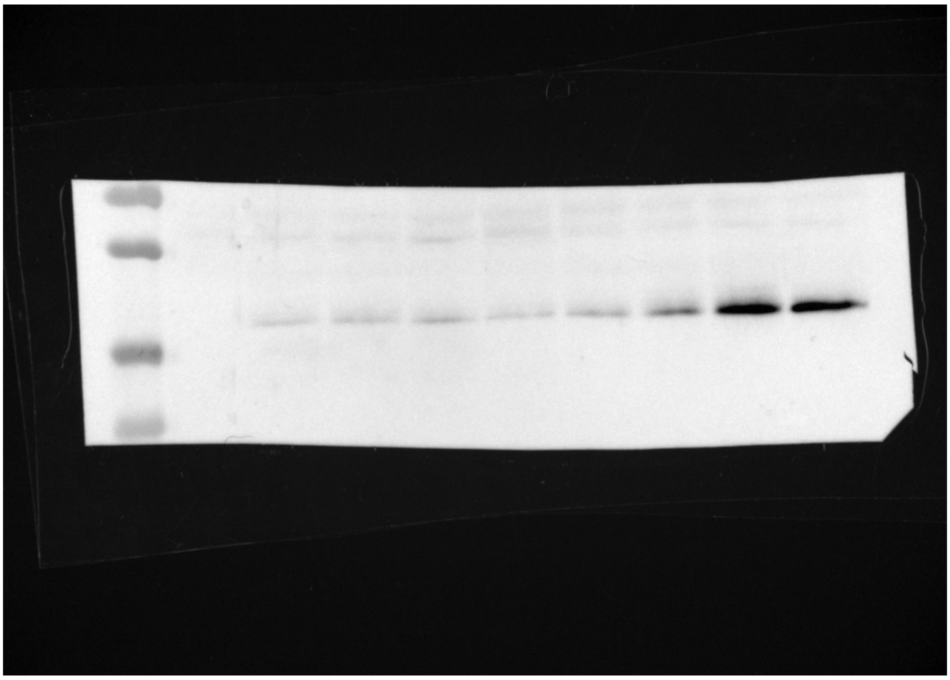

Ponceau Staining

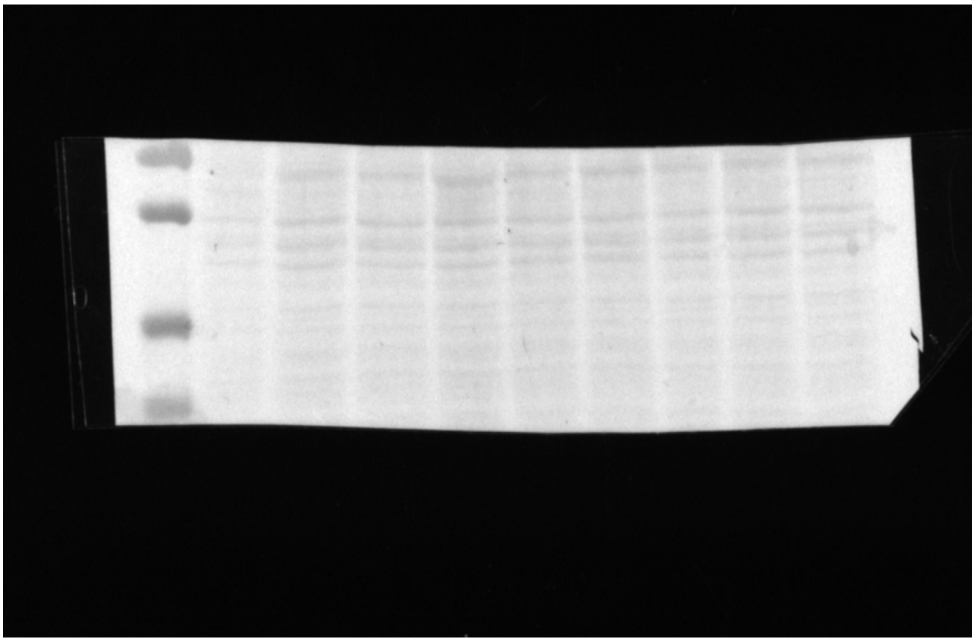

Fig-2, CX-43 Expression-Bands (EPI-7 Cells)

Propafenone

Marker Con DMSO CQ

10 20 30 40 50 μM

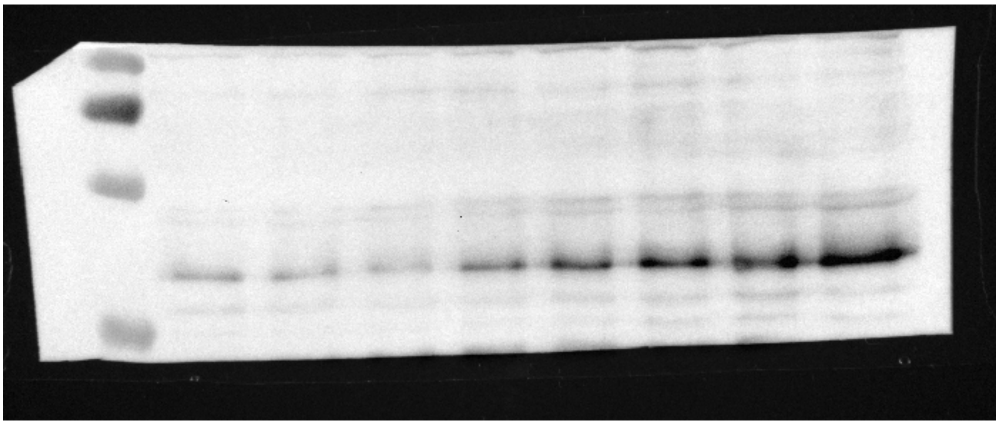

Ponceau Staining

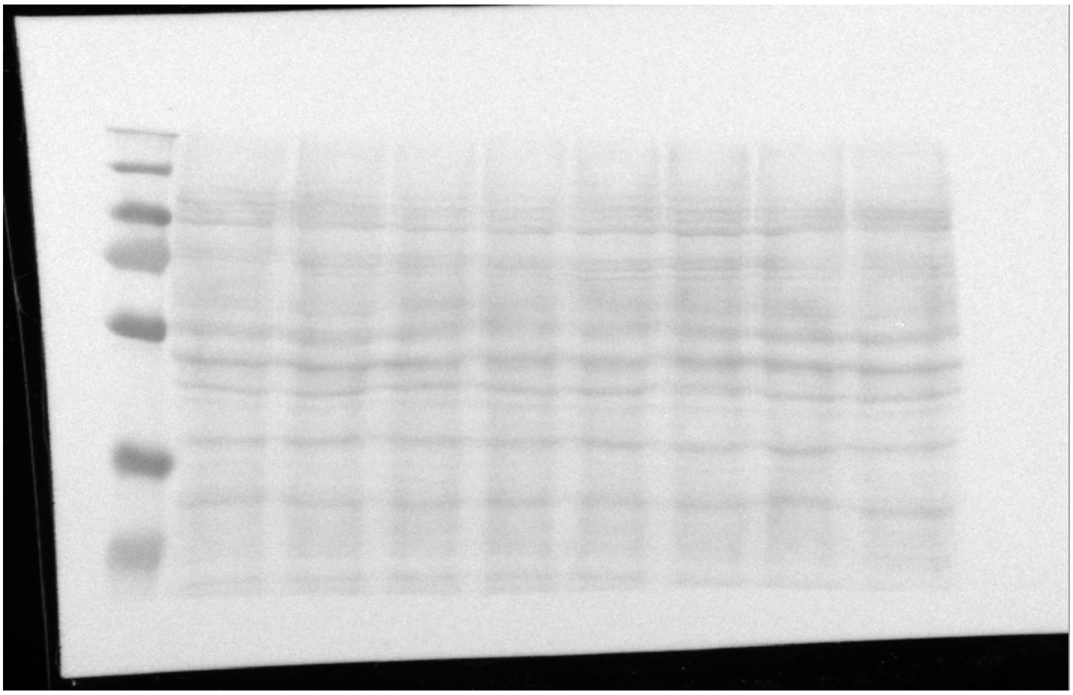

Fig-2, CX-43 Expression-Bands (END-2 Cells)

Marker Con DMSO

Propafenone

10 20 30 40 50 μM

CQ

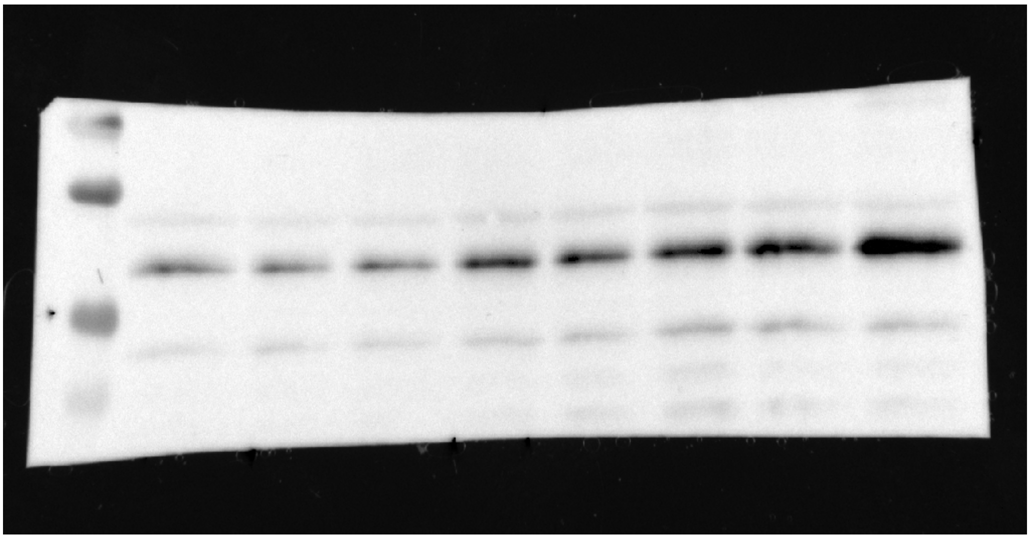

Ponceau Staining

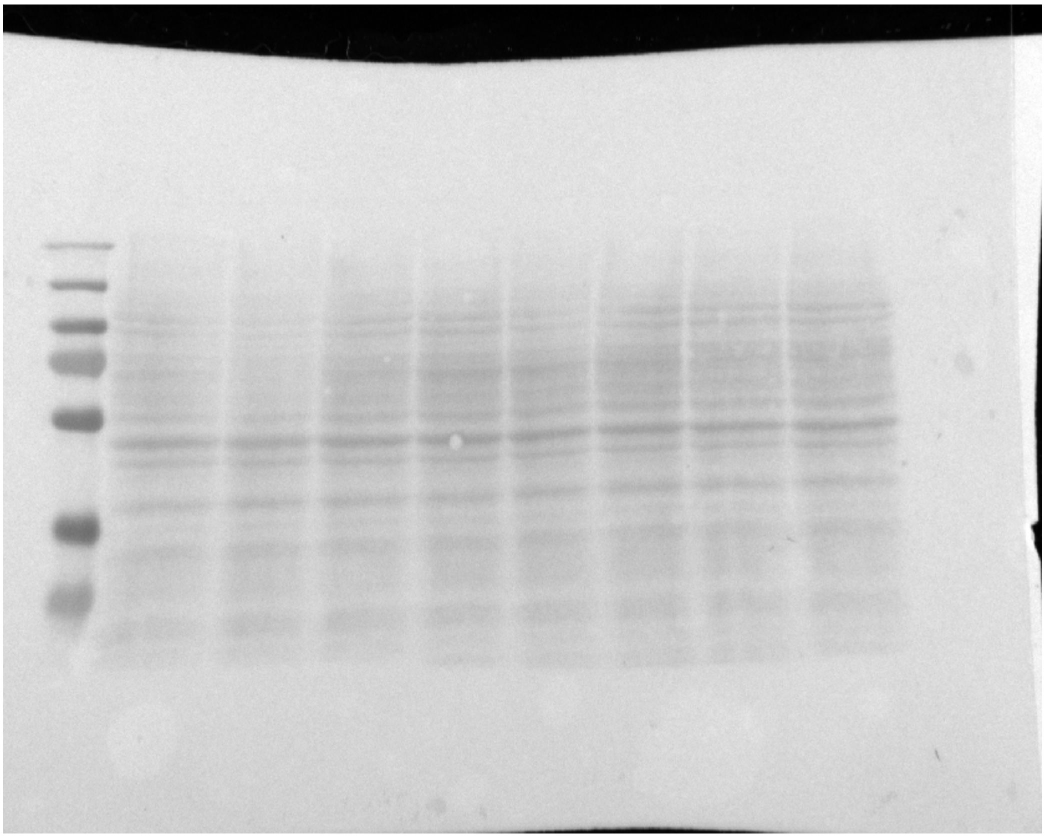

Fig-4, CX-43 Expression-Bands (EPI-7 Cells)

Marker Con

Propafenone (50 μM)

0 2 4 6 8 24 h

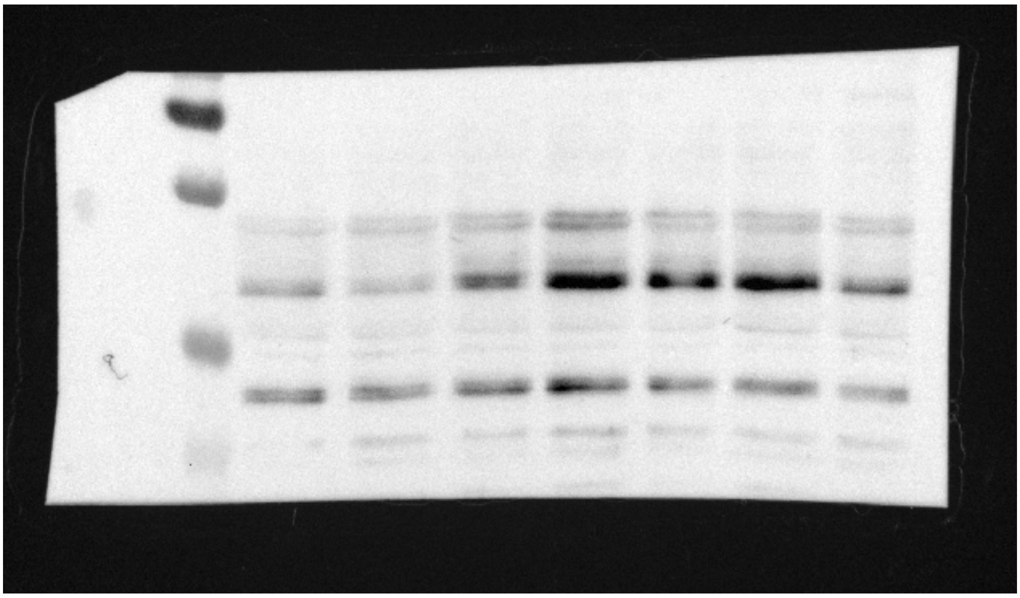

Ponceau Staining

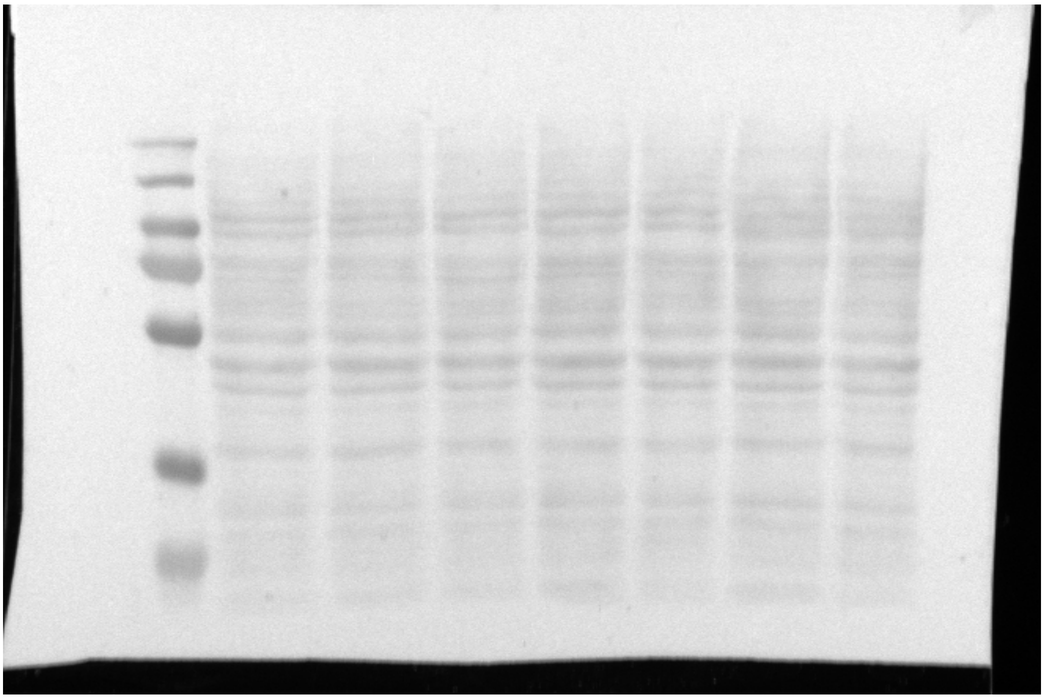

Fig-4, CX-43 Expression-Bands (END-2 Cells)

Marker Con Propafenone (50 μM)

0 2 4 6 8 24 h

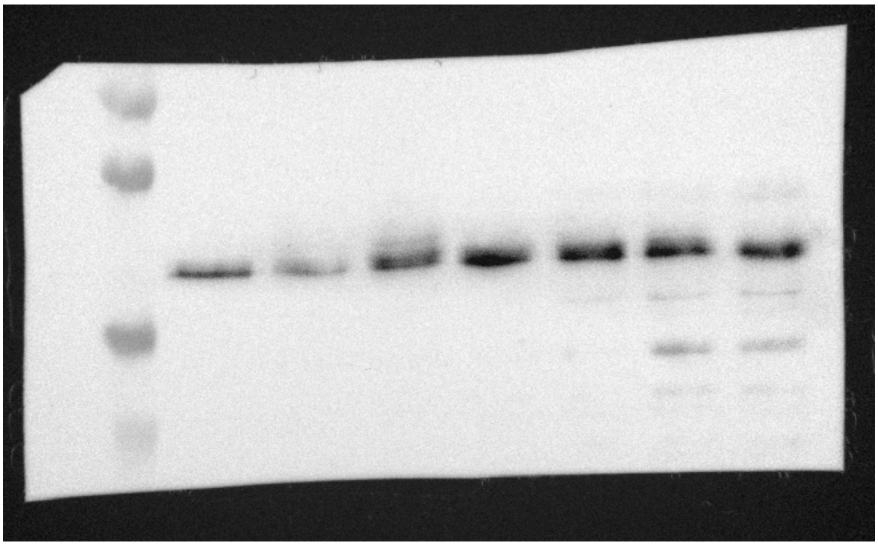

Ponceau Staining

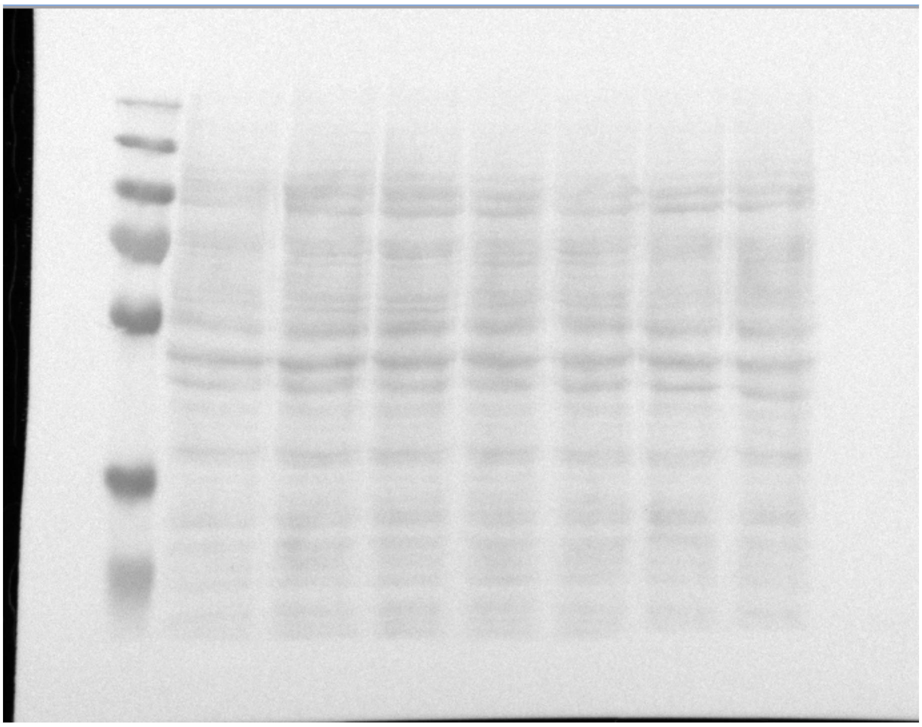

Fig-6, CX-43 Expression-Bands (EX-HEK Cells---Control)

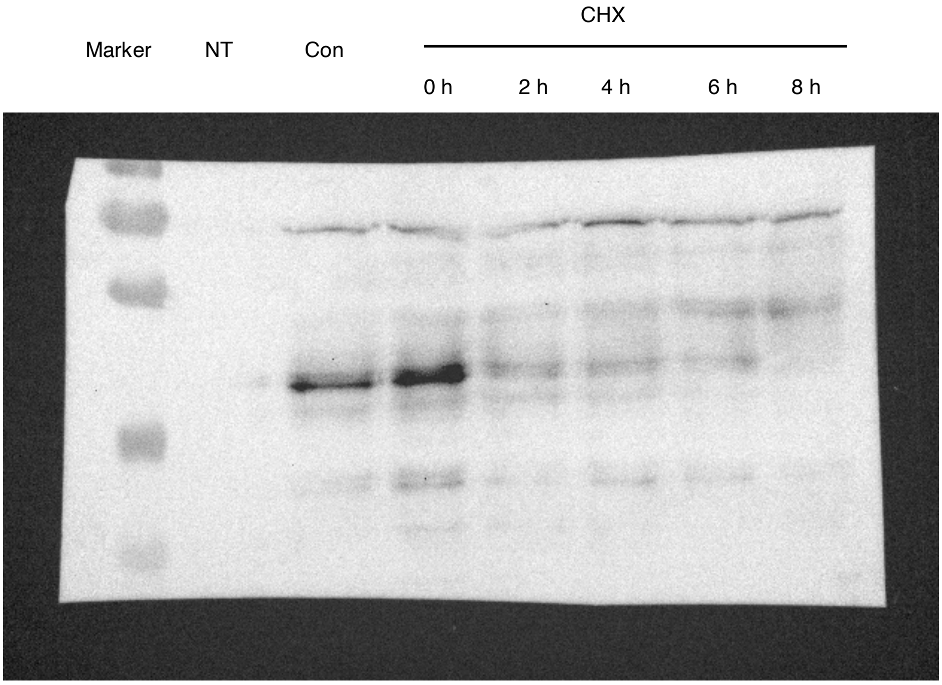

Ponceau Staining

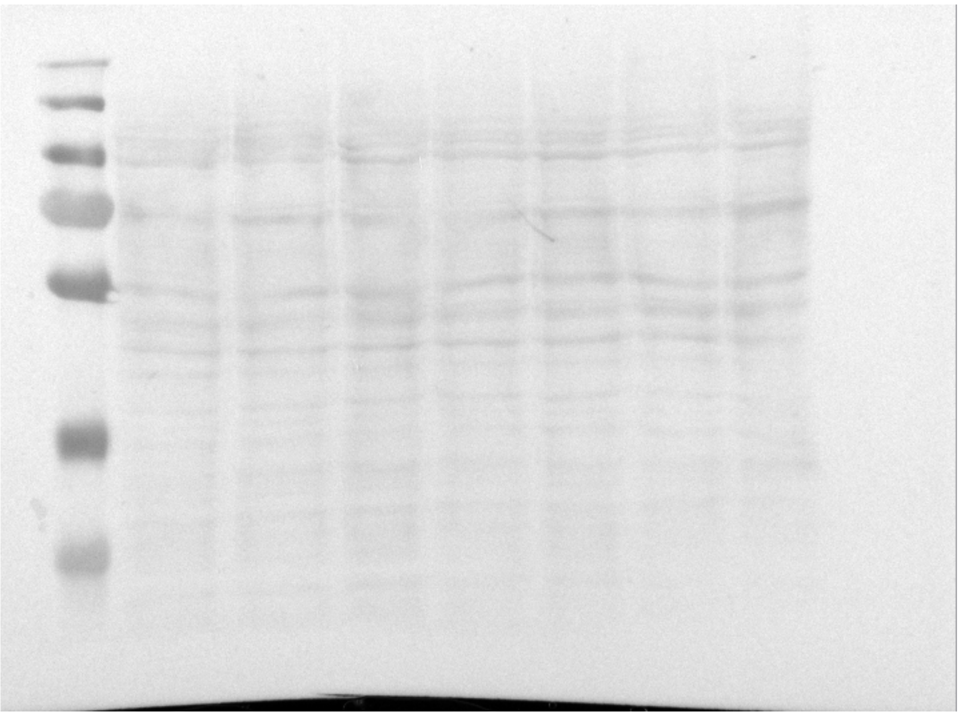

Fig-6, CX-43 Expression-Bands (EX-HEK Cells---Propafenone)

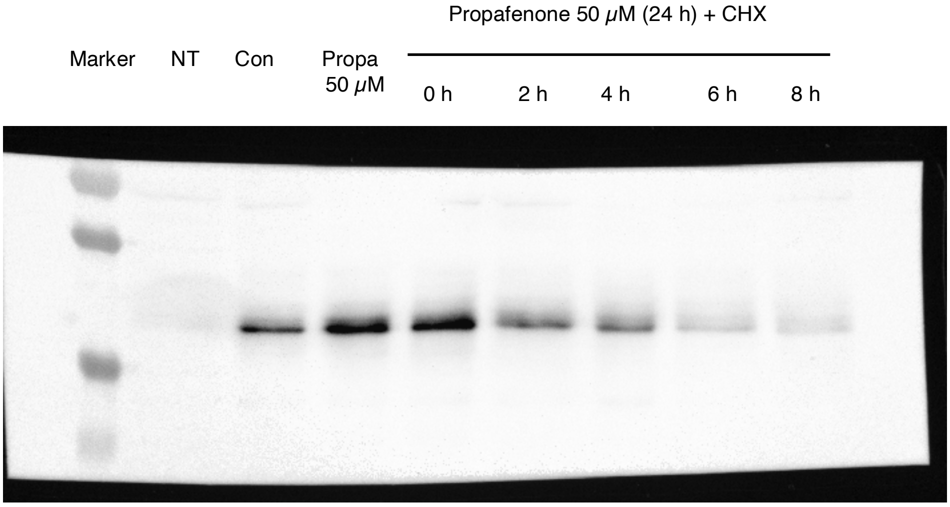

Ponceau Staining

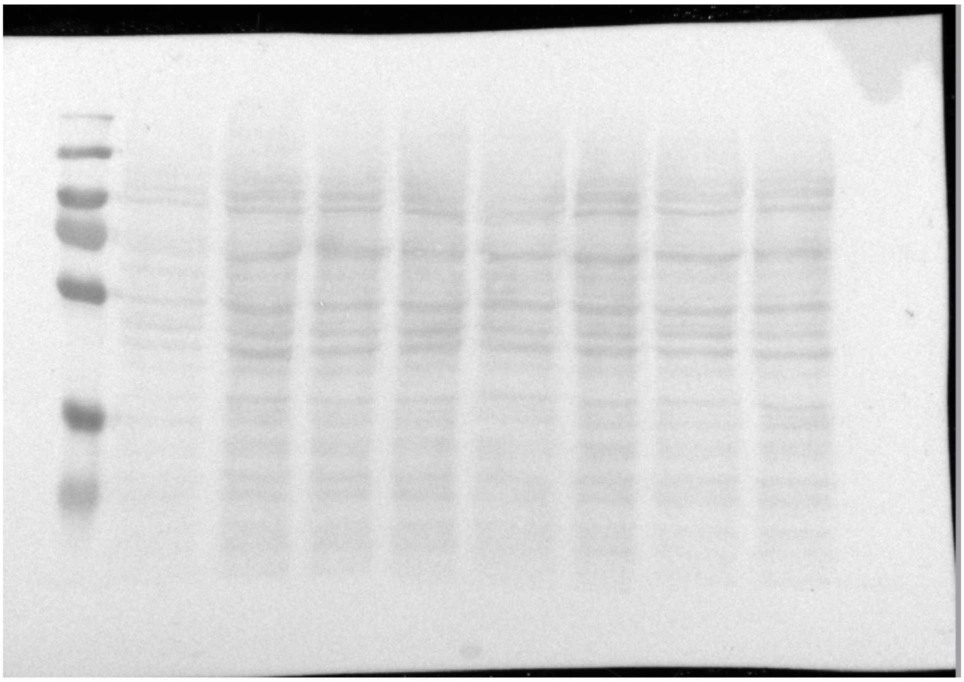

Fig-6, CX-43 Expression-Bands (EX-HEK Cells---CQ)

CQ 10 μM (24 h) + CHX

Marker NT Con CQ 10 μM 0 h 2 h 4 h 6 h 8 h

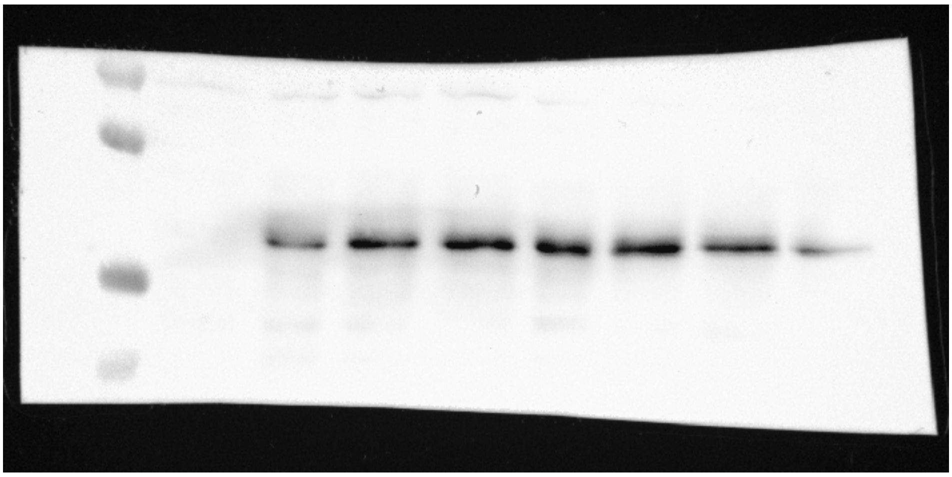

Ponceau Staining

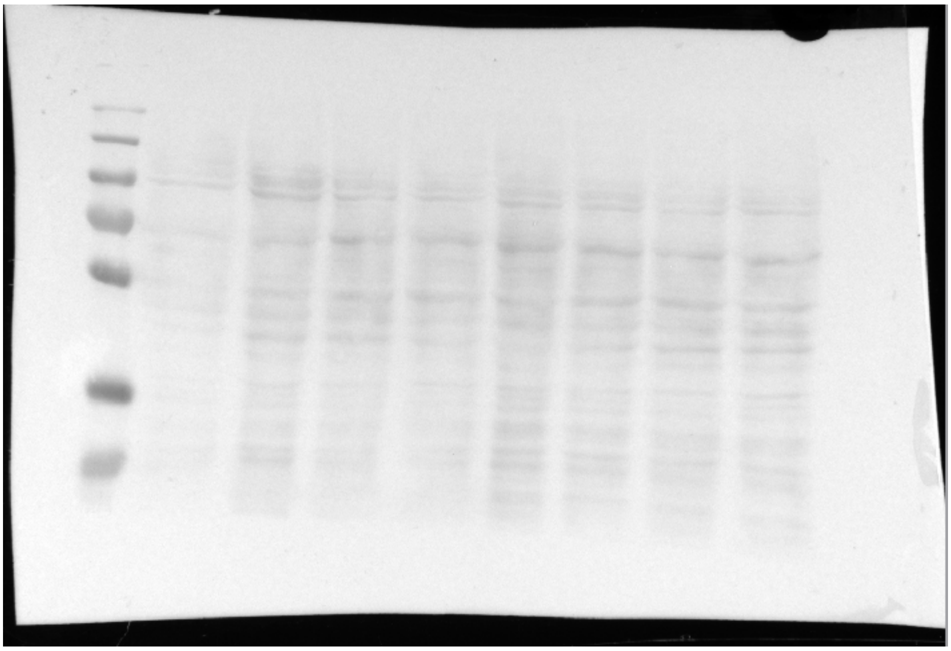

Fig-7, CX-43 Expression-Bands (EX-HEK Cells)

Marker NT Control DMSO Propafenone BFA Propafenone+BFA

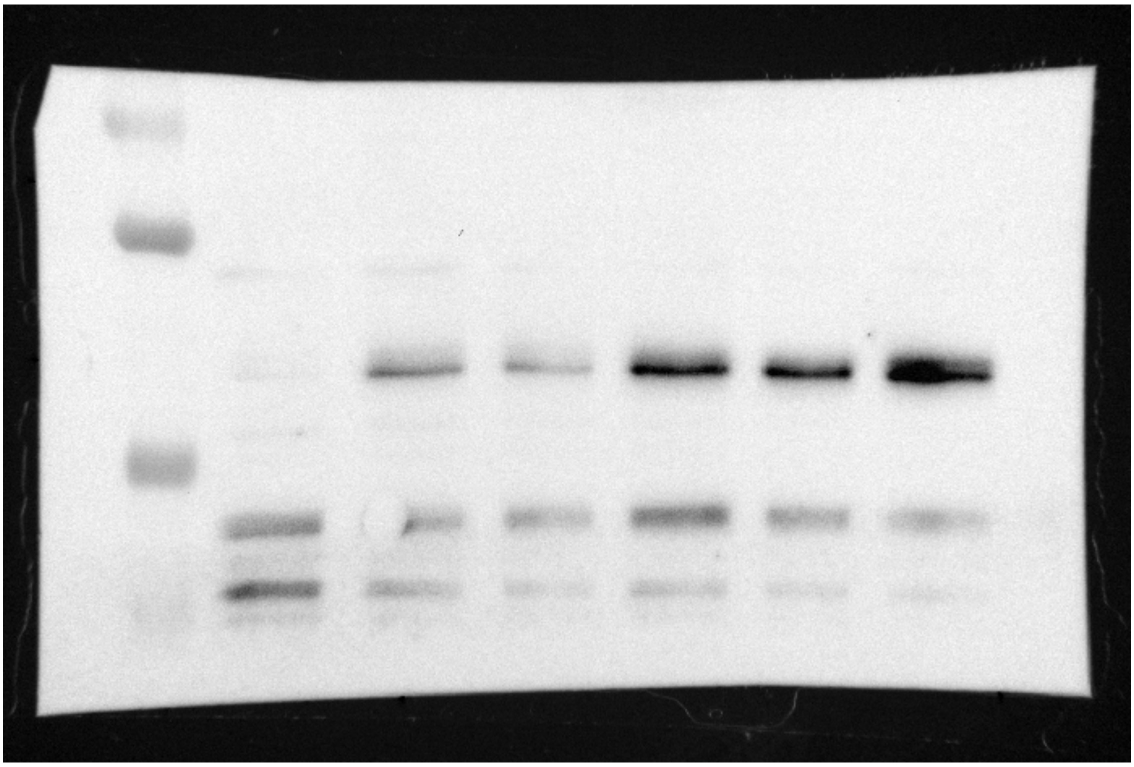

Ponceau Staining

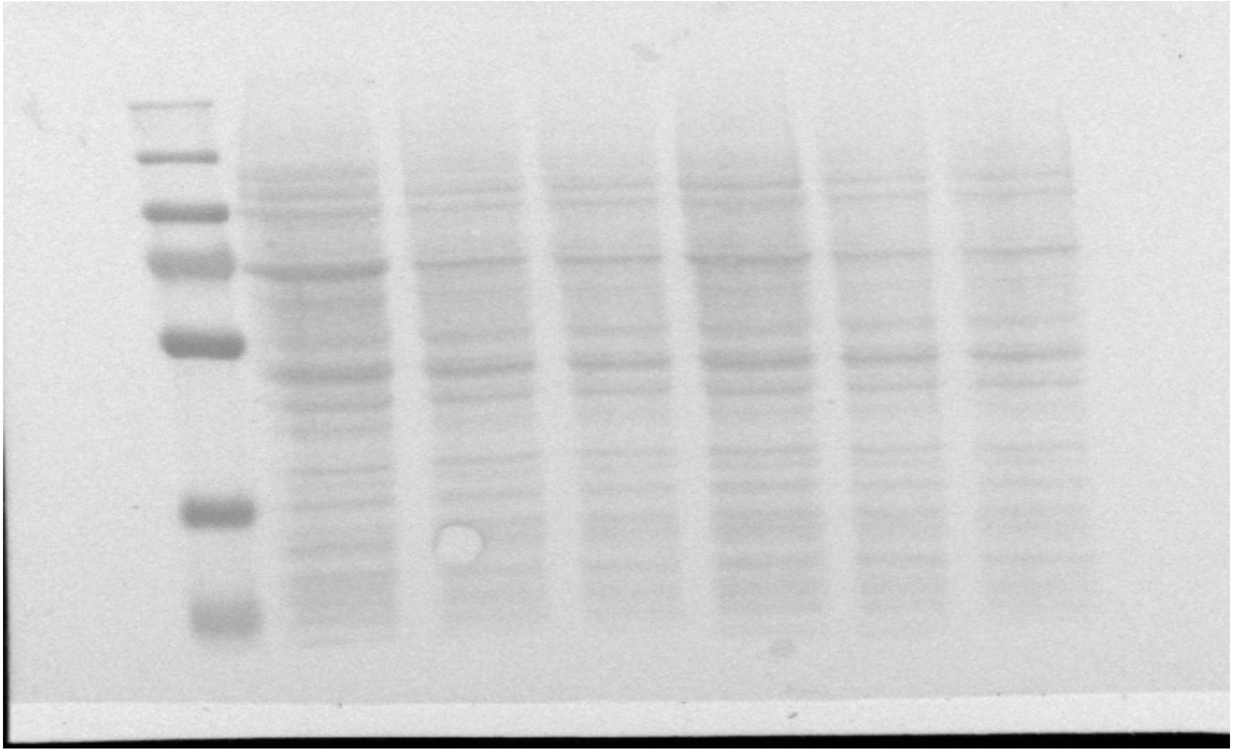

Supplement: Supplementary file 1 — Supplementary Material 1 [file 43440_2026_845_MOESM1_ESM.pdf]
