# Supplementary material for: Propafenone-mediated gap junctional uncoupling results from aberrant connexin-43 trafficking
Source: Pharmacol Rep. 2026 Mar 2;78(3):849–61. doi: 10.1007/s43440-026-00845-7 (PMC13275528; doi:10.1007/s43440-026-00845-7)

Full size blots supplementary figures S1 and S2

Original data

Fig-S1, ERS marker-Bands (IRE; Ex-HEK Cells)

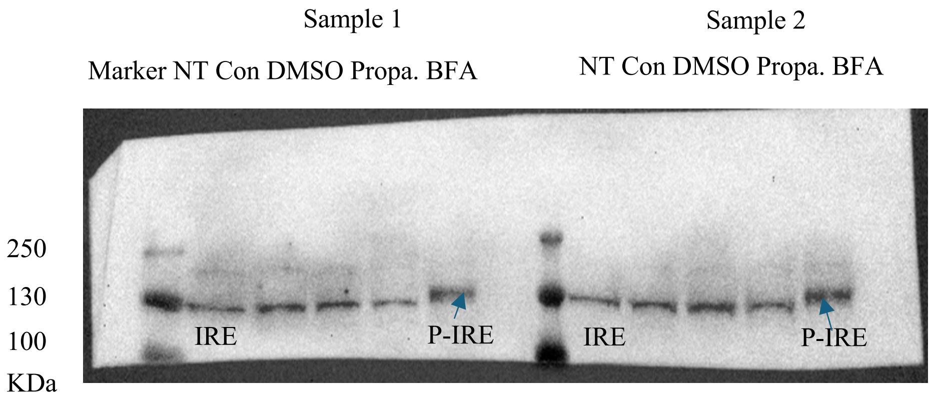

Ponceau Staining

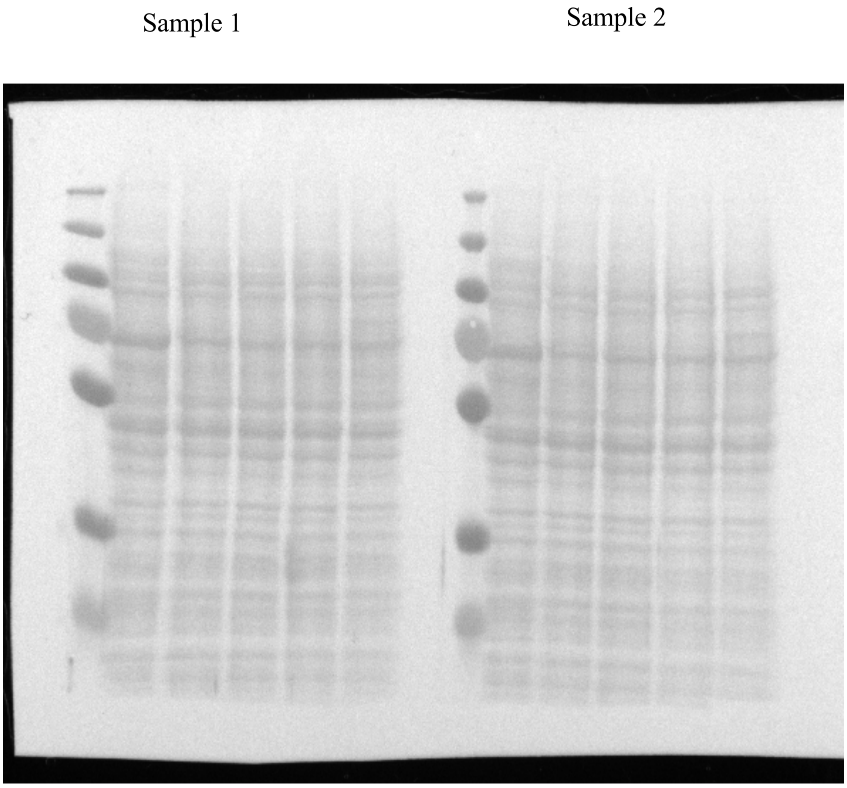

Fig-S1, ERS marker-Bands (PERK, Bip; Ex-HEK Cells)

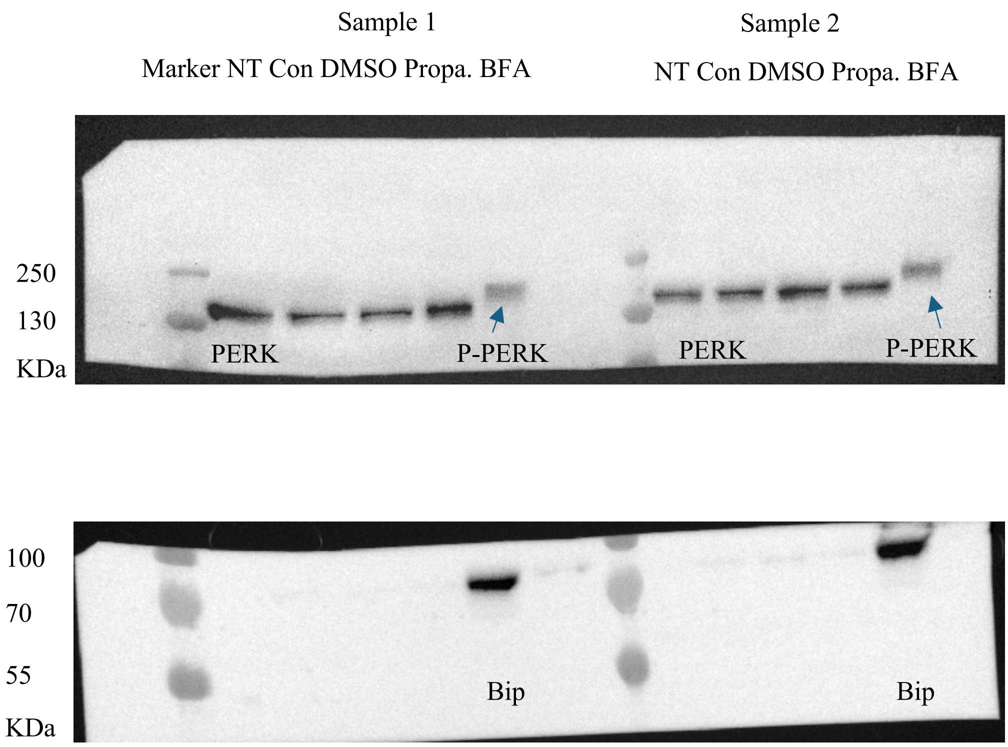

Ponceau Staining

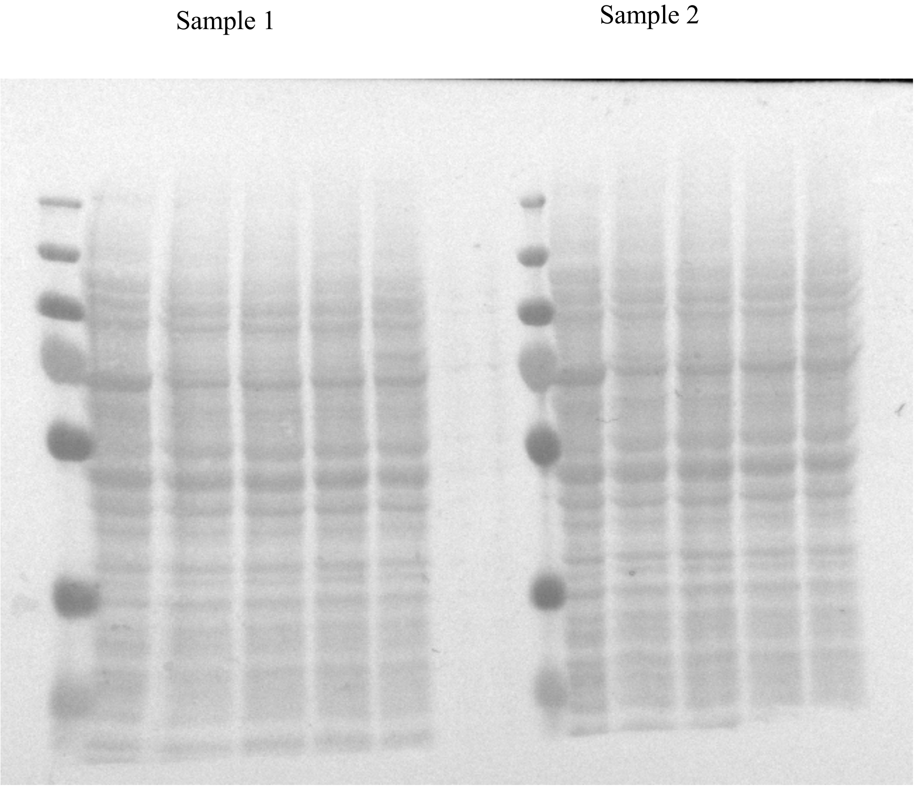

Fig-S1, ERS marker-Bands (Erol; Ex-HEK Cells)

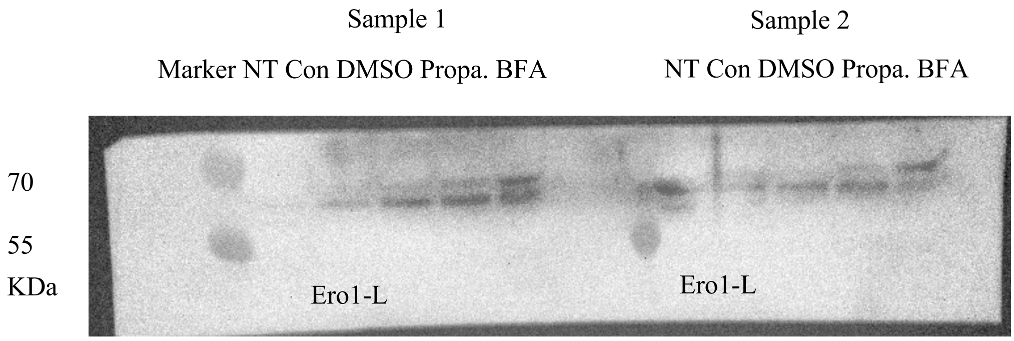

Ponceau Staining

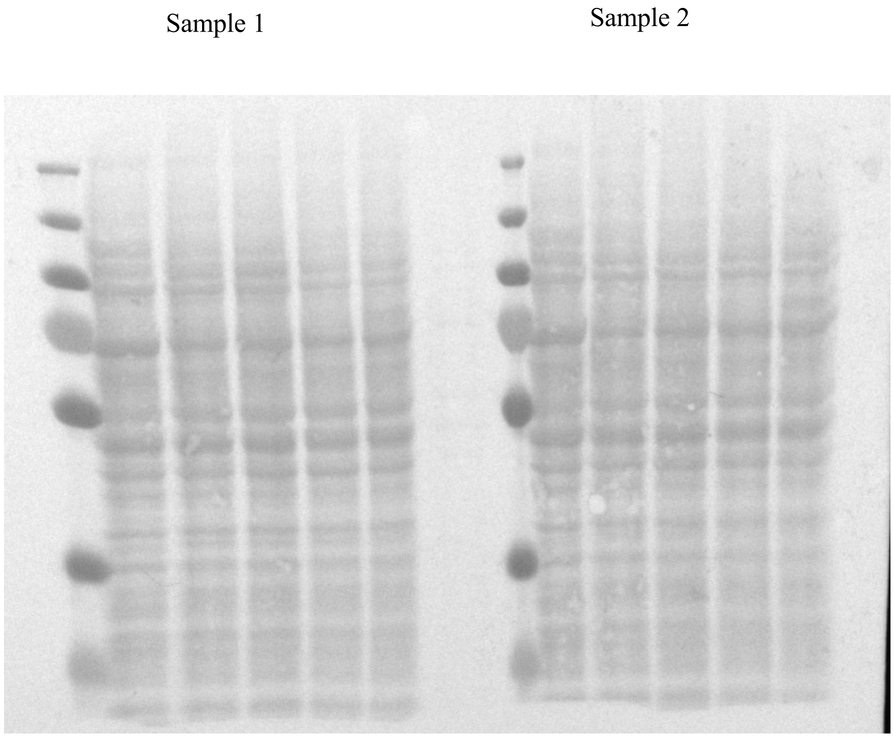

Fig-S1, ERS marker-Bands (CHOP; Ex-HEK Cells)

Sample 1 Sample 2

Marker NT Con DMSO Propa. BFA NT Con DMSO Propa. BFA

55

35

25

KDa

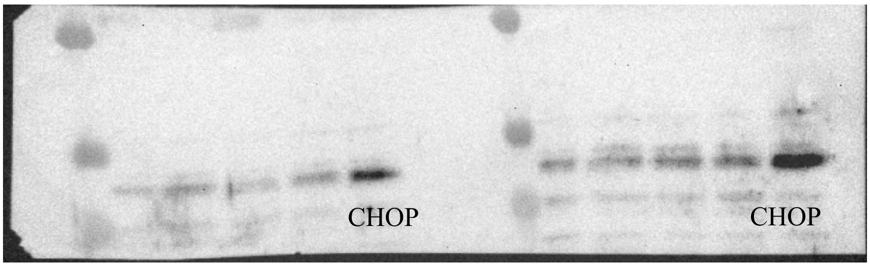

Ponceau Staining

Sample 1 Sample 2

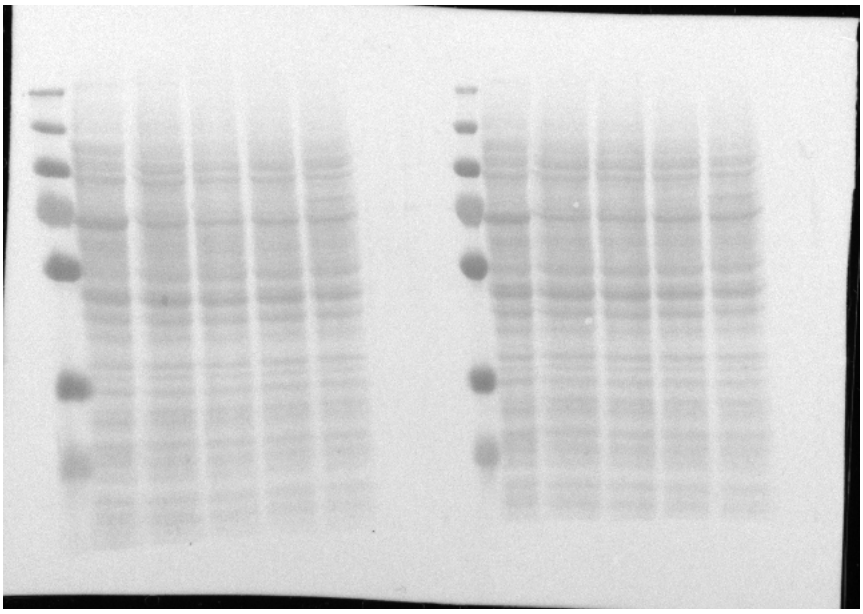

Fig-S1, ERS marker-Bands (PDI; Ex-HEK Cells)

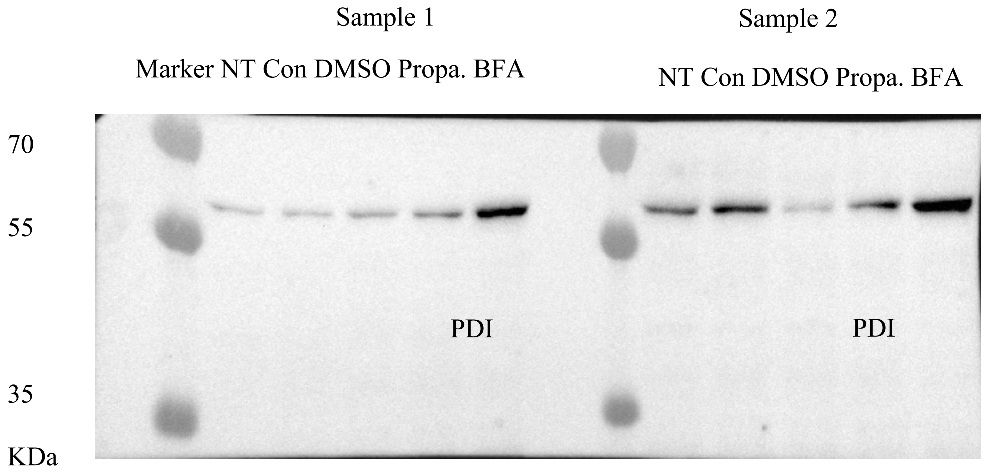

Ponceau Staining

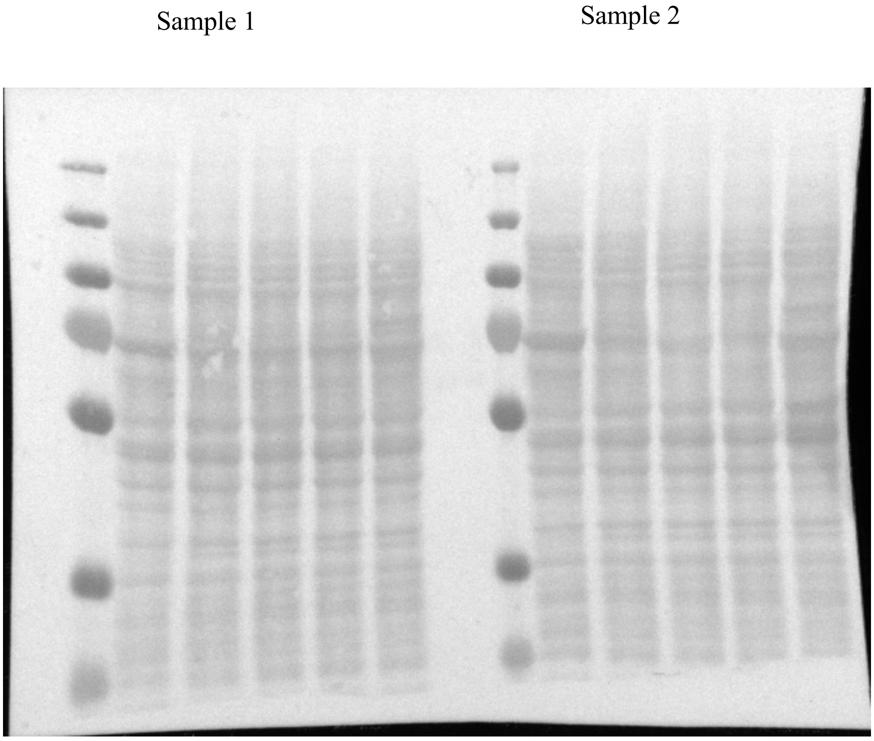

Fig-S2, Glu-α-tubulin -Bands (Ex-HEK Cells, EPI-7 Cells, END-2 Cells)

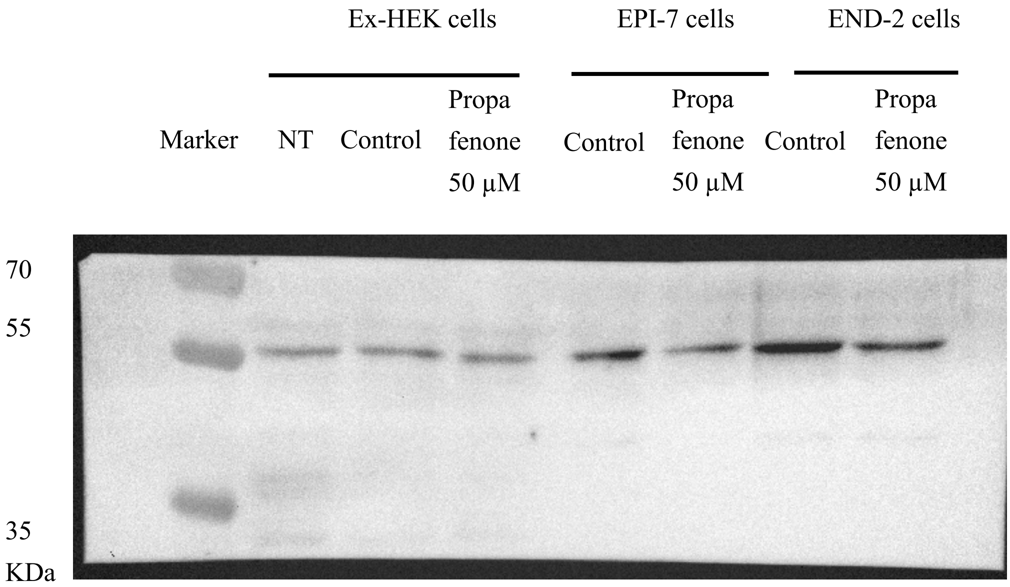

Ponceau Staining

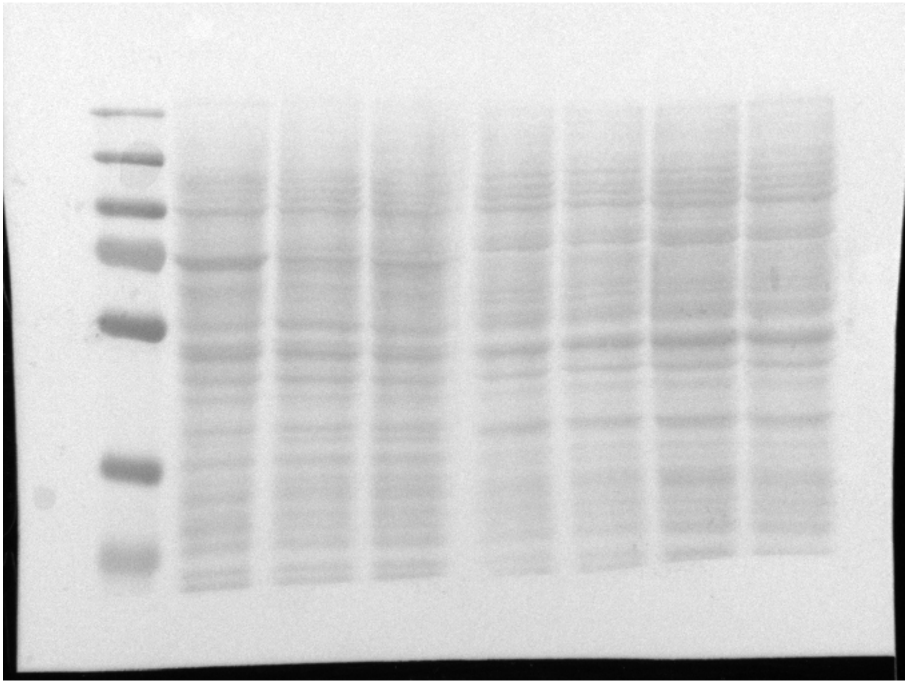

Supplement: Supplementary file 2 — Supplementary Material 2 [file 43440_2026_845_MOESM2_ESM.pdf]
