# Supplementary material for: Propafenone-mediated gap junctional uncoupling results from aberrant connexin-43 trafficking
Source: Pharmacol Rep. 2026 Mar 2;78(3):849–61. doi: 10.1007/s43440-026-00845-7 (PMC13275528; doi:10.1007/s43440-026-00845-7)
Supplement: Supplementary file 3 — Supplementary Material 3 [file 43440_2026_845_MOESM3_ESM.docx]

***Propafenone-Mediated Gap Junctional Uncoupling Results from Aberrant Connexin-43 Trafficking***

**Supplemental Figures**

**
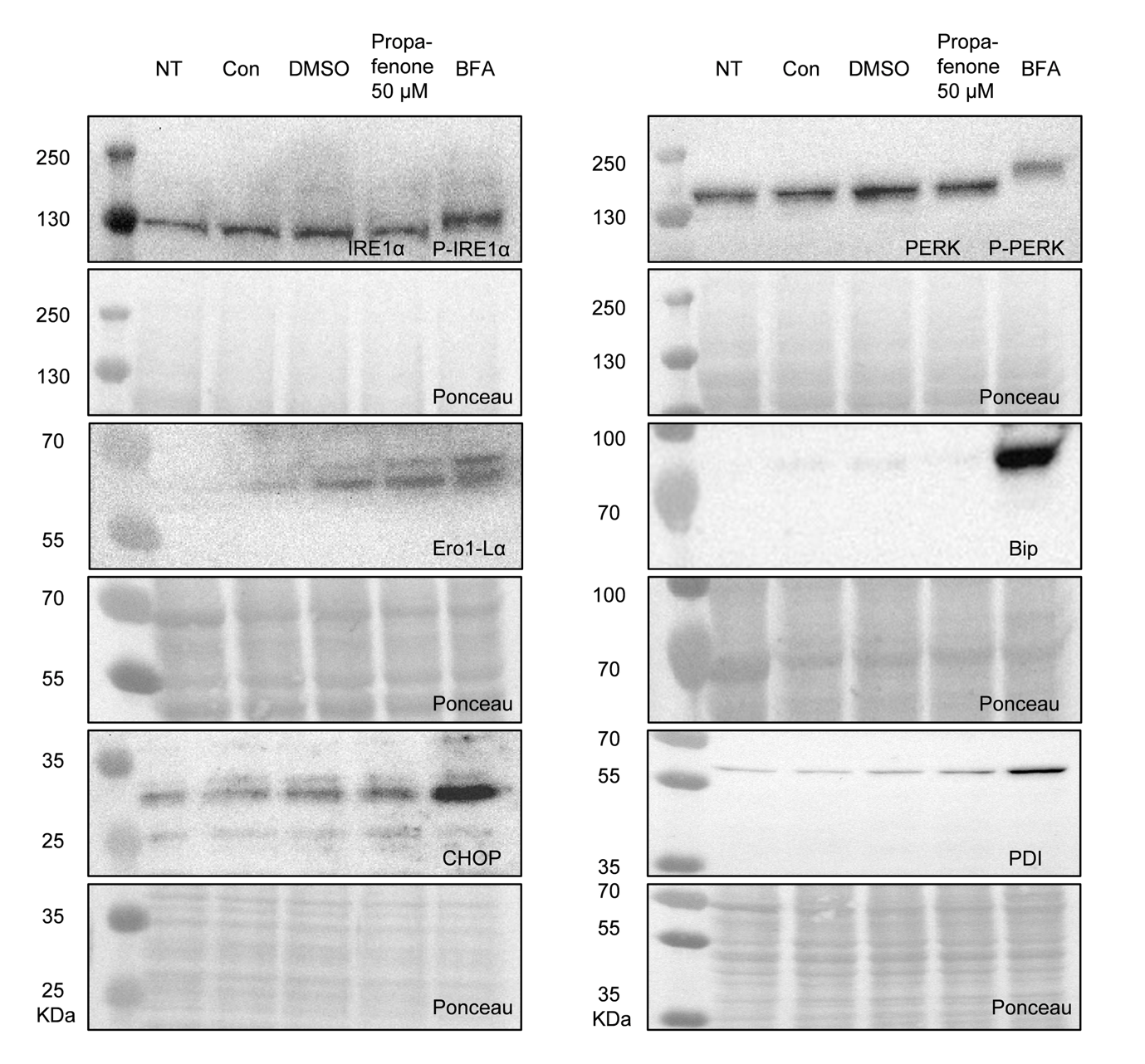
**

**Figure S1**: Propafenone, compared to positive control BFA, has minimal effects on ERS. Expression of 6 ERS markers (Inositol-requiring enzyme type 1α, endoplasmic reticulum oxidoreductin-1-Like α (Erol-Lα), CCAAT-enhancer-binding protein homologous protein (CHOP), PERK: protein kinase-like endoplasmic reticulum kinase pathways, immunoglobulin heavy chain binding protein (Bip), protein disulfide isomerase (PDI), in Ex-HEK cells and non-transfected HEK293 cells (NT). Con, control; DMSO (0.05%); BFA (2 µg/mL).

**
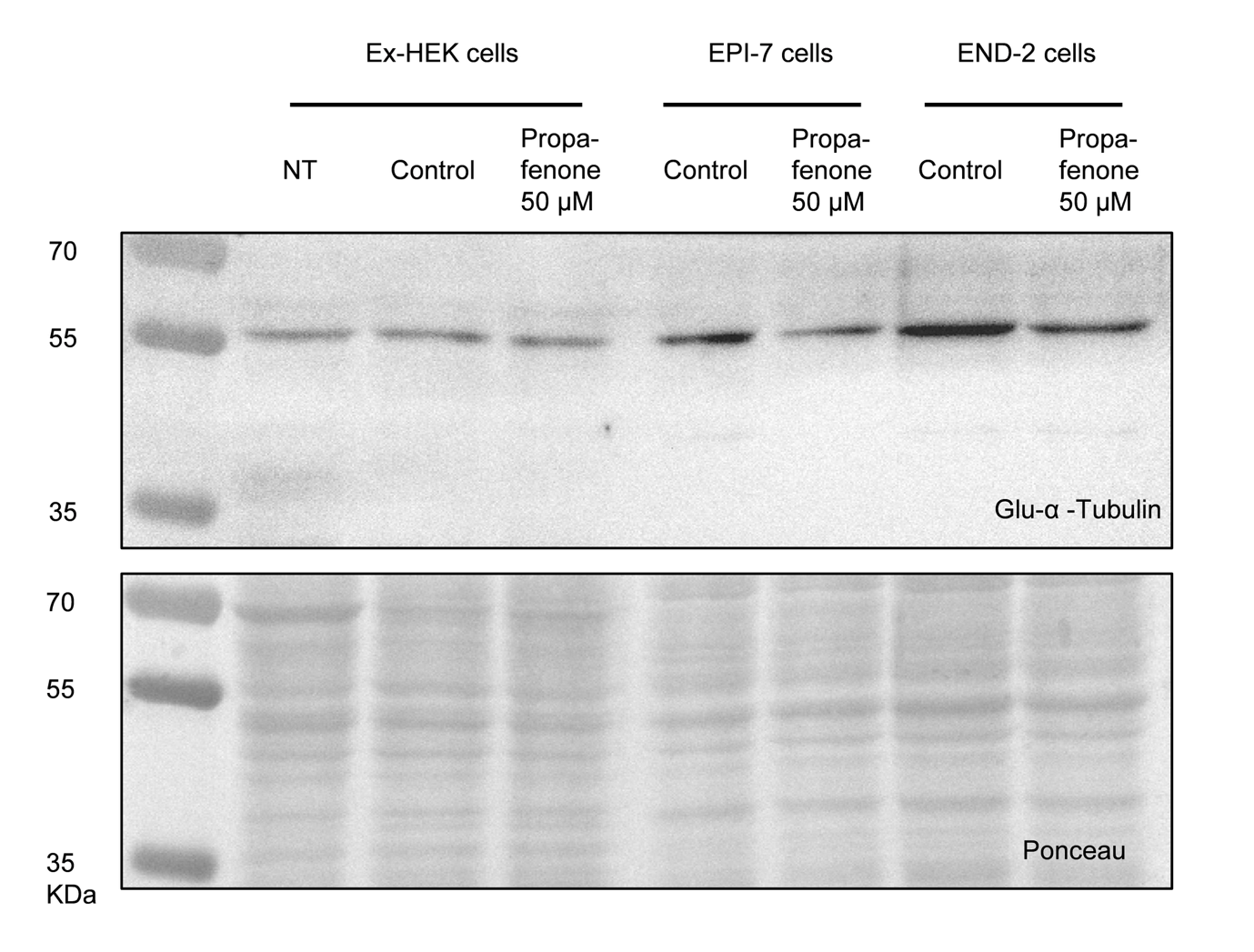
**

**Figure S2**: Propafenone does not increase Glu-α-tubulin expression levels in Ex-HEK, EPI-7 and END-2 cells. NT, non-transfected HEK293 cells.
